# Experimental evidence of a biological soil crust degradation climate warming amplification feedback

**DOI:** 10.1038/s43247-026-03874-5

**Published:** 2026-09-12

**Authors:** William K. Smith, Miguel L. Villarreal, Cara Lauria, Willliam A. Rutherford, Stefanie Herrmann, Victoria Scholl, Armin Howell, Mostafa Javadian, Fujiang Ji, Fangyue Zhang, Matthew A. Burgess, Raymond Kokaly, Benjamin Poulter, Sasha C. Reed

**Affiliations:** 1 School of Natural Resources and the Environment, University of Arizona, Tucson, AZ USA; 2 U.S. Geological Survey, Western Geographic Science Center, Moffett Field, CA USA; 3 U.S. Geological Survey, Southwest Biological Science Center, Moab, UT USA; 4 U.S. Department of Interior, Bureau of Land Management, Arizona State Office, Tucson, AZ USA; 5 Arizona Institute for Resilience, University of Arizona, Tucson, AZ USA; 6 U.S. Geological Survey, National Uncrewed Systems Office, Geosciences and Environmental Change Science Center, Denver, CO USA; 7 U.S. Geological Survey, Geology, Geophysics, Geochemistry Science Center, Denver, CO USA; 8 Spark Climate Solutions, San Francisco, CA USA

**Keywords:** Ecosystem ecology, Climate-change ecology

## Abstract

Biological soil crusts – photosynthetic communities of cyanobacteria, lichens, and/or mosses living on soil surfaces – represent about 12% of the global land surface, yet their role in climate mitigation remains uncertain. Here we address this knowledge gap by integrating multiscale remote sensing techniques within a unique long-term climate manipulation experiment. Two decades of in situ climate warming and changes in monsoonal rainfall drove a major shift in biocrust community composition, including a ~ 27% decrease in fractional coverage of late-successional mosses and a ~ 36% increase in fractional cover of early-successional, lightly-pigmented cyanobacteria. This community shift, in-turn, resulted in a ~ 6.3% reduction in photosynthetic potential, ~14.1% increase in surface brightness, ~15.2% decrease in surface moisture, and a ~ 1.5% or ~0.3 °C increase in surface temperature under clear sky daytime conditions for every 10% increase in the relative cover of lightly-pigmented cyanobacteria. Our findings are evidence of a biocrust degradation climate warming amplification feedback, whereby climate warming drives biocrust functional degradation and a net reduction in climate mitigation potential, which further drives warming. This apparent warming amplification feedback is currently missing from process-based models, and thus current climate projections might underestimate the rate of climate warming across global drylands.

## Abstract (web summary)

Climate warming and changes in monsoonal rainfall resulted in a biocrust community composition shift from late-successional mosses to early-successional, lightly pigmented cyanobacteria , resulting in a substantial reduction in photosynthetic potential and net increase in local surface temperatures, based on remote sensing with a long-term dryland experiment in Castle Valley, Utah, USA.

## Introduction

Drylands – hyperarid, arid, semiarid, and dry subhumid ecosystems – are defined by their aridity and together represent Earth’s largest terrestrial biome, accounting for ~45% of the planet’s land surface^1^. The climatic characteristics of drylands, coupled with the relatively low fertility of their soils, impose limitations on dryland biota^2,3^. Because of these constraints, dryland plant cover is typically sparse relative to other biomes, and thus these systems characteristically maintain a diverse photosynthetic biological soil crust (biocrust) community in the interspaces and below the canopy of vascular plants^2,4,5^. These autotrophic soil communities consist of cyanobacteria, lichens, and/or mosses and occur upon and within the top few cm of soils and can be the dominant living cover found in these regions^6^. Because biocrusts mediate almost all inputs and exports from dryland soil surfaces, they comprise a “critical zone”, heavily influencing soil fertility, soil stability, ecosystem carbon uptake, plant diversity, local and regional hydrology, albedo, energy balance, and ecosystem biogeochemical cycling^4,5,7–10^.

Biocrusts help regulate biogeochemical cycling and improve soil fertility by fixing carbon dioxide (CO_2_) and nitrogen (N_2_) in parts of ecosystems where plants are absent or inactive^11–13^. Biocrusts greatly increase soil stability and protect against erosion and atmospheric dust emission by binding soil minerals together^10,14^. Additionally, biocrusts impact local and regional hydrology by reducing runoff and improving soil moisture storage^8,15^. Biocrusts also take up substantial amounts of CO_2_, with the amount and fate of their fixed carbon varying among different biocrust organisms and successional states^16^. Finally, biocrusts influence local and regional energy balance by altering surface roughness and albedo. Late-successional biocrust communities, often dominated by lichens and mosses, increase surface roughness and reduce albedo compared to early-successional cyanobacteria biocrusts or bare soil that is absent of biocrust^15,17,18^. Taken together, the emerging evidence is that biocrusts play a major role in dryland carbon, water, and energy cycling and that they cover vast expanses of the land surface – ~30% of global drylands or ~12% of the global land surface^10^, underscoring the importance of this relatively poorly understood functional group from local to global scales^19^.

Simultaneously, evidence is emerging that the health and functioning of biocrusts are highly sensitive to both land use and climate change e.g., refs. ^20,21^. Experimental warming and altered precipitation regimes have been found to drive dramatic degradation of biocrust communities, characterized by losses of late-successional mosses, lichens, and soil stability and expansion of early-successional light cyanobacteria cover with reduced functional capacity^20–23^. Across the western U.S., drylands have already experienced increasing temperatures and changing amounts, frequency, and intensity of precipitation^24,25^, with largely unknown consequences for biocrust community composition and overall ecosystem function across space and time scales^26,27^. Adding urgency, warming and changes in precipitation regimes are projected to accelerate over the coming decades^28,29^. Yet, our understanding of how climate change is affecting biocrust community composition and the processes they regulate remains far from complete and comparatively understudied.

Remote sensing has played a defining role in advancing our understanding of vegetation-climate interactions at regional and global scales, affording the ability to upscale findings from the site to the entire terrestrial biosphere^30^. In particular, remote sensing has been applied to study in-depth the climate mitigation potential of vascular plants, e.g., their ability to absorb CO_2_ and cool local surface tempertures, across diverse ecosystems^31–34^. Yet, a comprehensive assessment of the climate mitigation potential of biocrusts is still lacking, despite their established importance in dryland carbon, water, and energy cycling and their vast expanses, due in part to the inherent complexity of biocrust community composition and the challenges associated with quantifying biocrust function at the species scale across large areas^35^. Limitations of currently available aircraft and satellite remote sensing platforms in their ability to capture the complex sub-pixel scale community composition of biocrusts have compounded these challenges^30,36^.

**Fig. 1:**
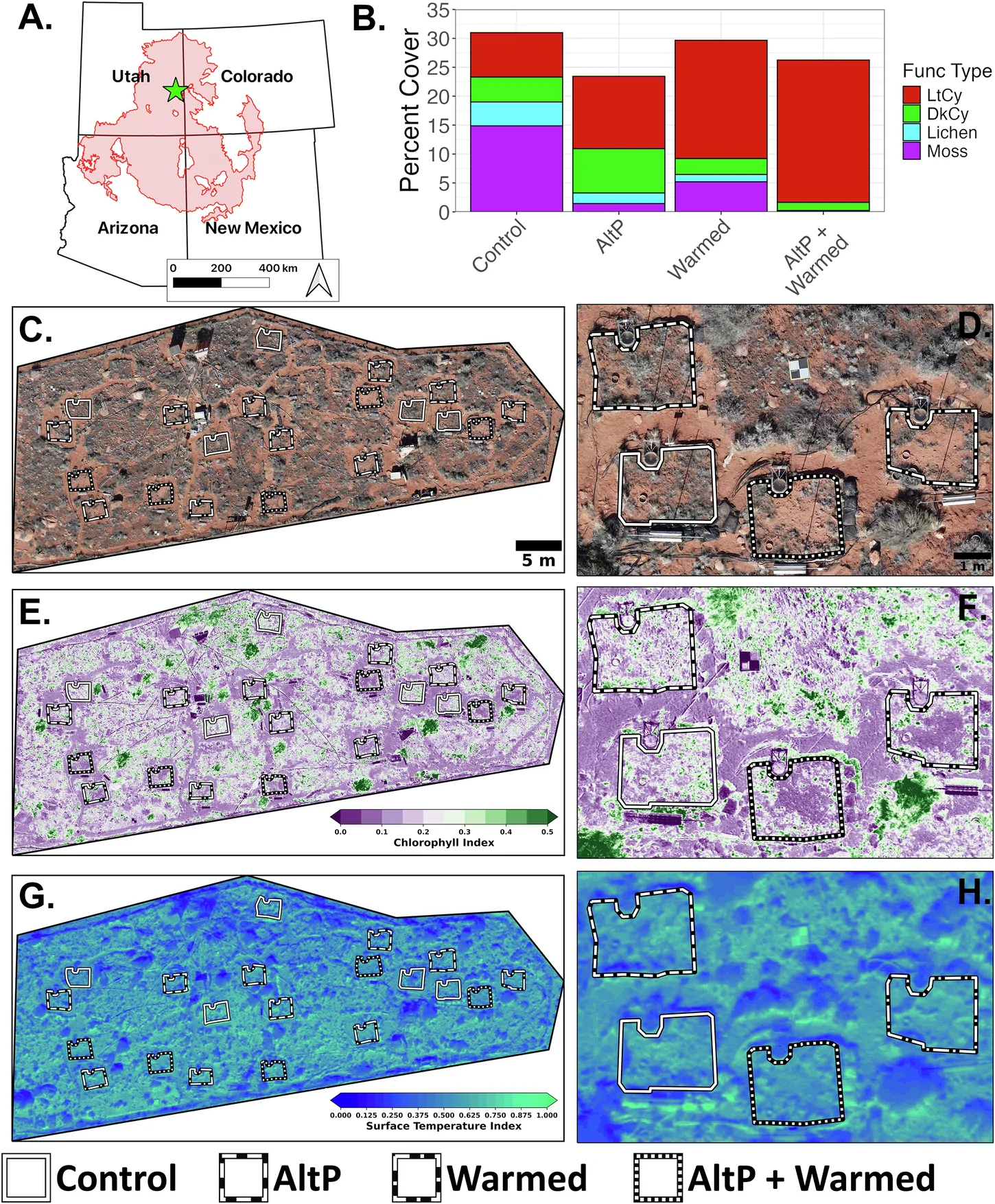
The long-term biocrust climate manipulation experiment located near Castle Valley, Utah (38.67° N, 109.42° W) on the Colorado Plateau. Map showing location of the study site (green star) and Colorado Plateau Ecoregion (red polygon) spanning U.S. states Colorado, Utah, Arizona, and New Mexico (**A**). Bar plot showing the percent cover of the major biocrust functional types (BFTs; lightly pigmented cyanobacteria (LtCy), darkly pigmented cyanobacteria (DkCy), lichen, and moss) and the dominant shift from mosses in the control plots to lightly pigmented cyanobacteria in the warmed plots (**B**). Uncrewed aerial system (UAS)-based RGB image of the study site (**C**) and a zoomed-in aerial view of the easternmost plots representing the four experimental treatments (treatments and associated line types are listed below the figure: Control, Altered Precipitation (AltP), Warmed, and AltP and Warmed (AltP + Warmed treatments) (**D**). UAS-based Chlorophyll Index image of the study site (**E**) and a zoomed-in aerial view of the easternmost plots representing the four experimental treatments (**F**). UAS-based Surface Temperature Index image of the study site (**G**) and a zoomed-in aerial view of the easternmost plots representing the four experimental treatments (**H**).

Here we address this knowledge gap by integrating multiscale remote sensing techniques within an established in situ dryland altered climate experiment on the Colorado Plateau of the western U.S. (Fig. 1, S1). This long-term (20-year) experimental manipulation includes altered precipitation (increased precipitation frequency) and warming treatments (4 °C above ambient) and has captured major climate change-driven state shifts in biocrust community composition^20–22,37^. In particular, warming alone was found to drive a major state shift in biocrust community composition, transitioning the community from a mixture of late-successional, darkly pigmented cyanobacteria, lichens, and mosses to dominance by the early-successional, lightly pigmented cyanobacteria biocrust functional type (BFT)^20^. Here, through the integration of novel optical and thermal infrared remote sensing techniques at both the BFT- and plot-scale, we aimed to assess the impacts of climate change-driven shifts in biocrust community composition on biocrust climate mitigation potential, represented by the following functional indices: chlorophyll index (e.g., photosynthetic potential), surface moisture index, surface brightness index, and surface temperature index. We ask two major questions: (1) how do different BFTs contribute to these key functional indices?; and (2) what is the impact of the climate change-driven shifts in biocrust community composition on these functional indices?

## Results

### Biocrust functional-group level indices

**Fig. 2:**
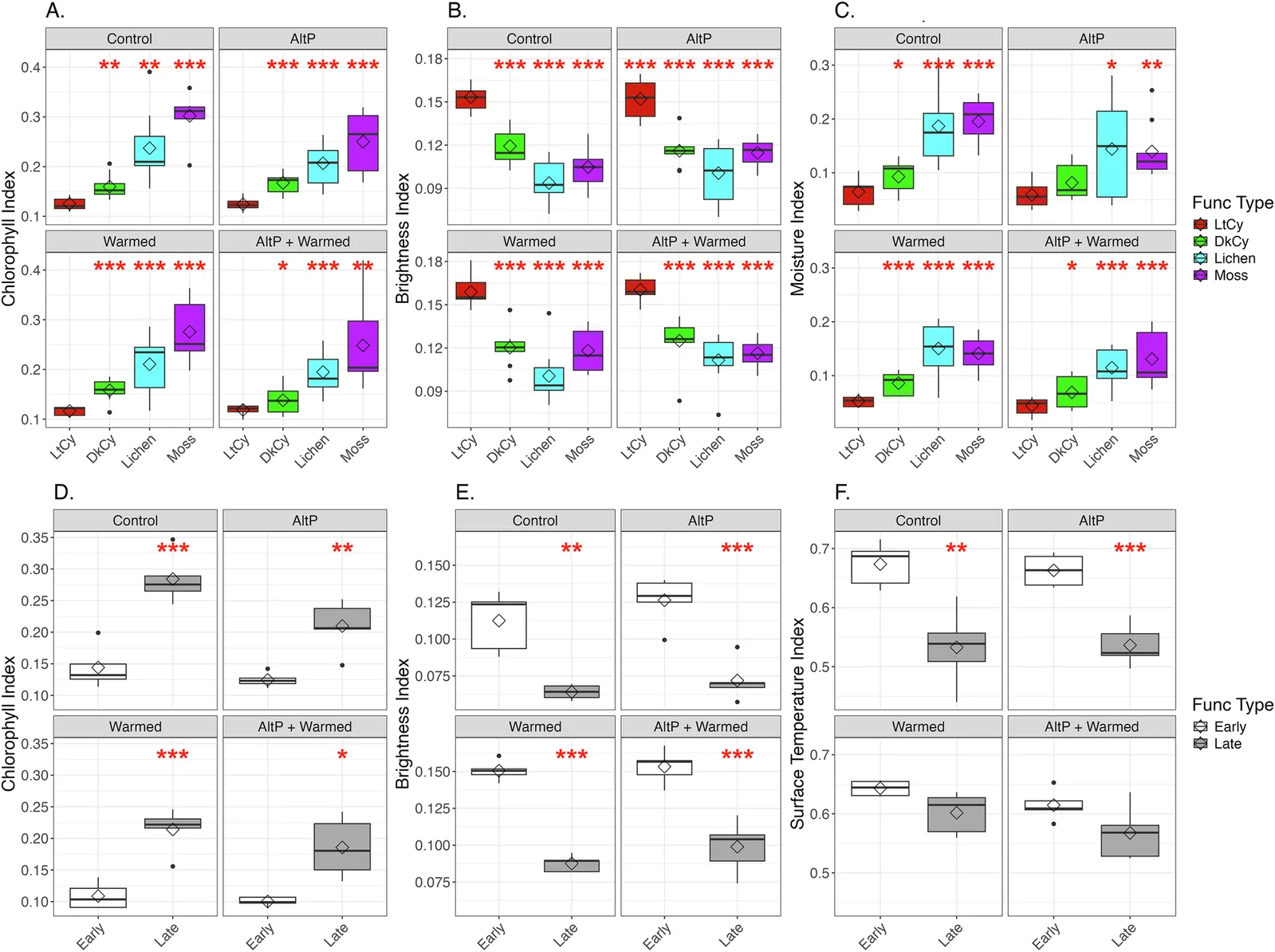
Differences in remotely sensed functional traits across biocrust functional types (BFTs). The top row shows field spectroradiometer ground-based estimates of chlorophyll (**A**), brightness (**B**), and moisture (**C**) indices by experimental treatment and across biocrust functional types (BFTs). The bottom row (**D**–**F**) shows uncrewed aerial systems (UAS) estimates of chlorophyll (**D**), brightness (**E**), and temperature (**F**) indices by experimental treatment and across early-successional (Early) and late-successional (Late) biocrust communities. Experimental treatments are shown in each panel and include control (Control), altered precipitation (AltP), warming (Warmed), and altered precipitation and warming combined (AltP + Warmed) treatments. BFTs are shown in different colors and include lightly pigmented cyanobacteria (LtCy), darkly pigmented cyanobacteria (DkCy), lichen (Lichen), and moss (Moss). Statistical significance is shown as *p < 0.05; **p < 0.01; and ***p < 0.001.

Based on isolated spectra collected using a field spectroradiometer (Fig. S2), we found a clear gradient in remotely sensed functional traits across major biocrust functional types (BFTs), and these differences generally persisted across experimental treatments (Fig. 2A–C). Mosses had the highest chlorophyll [median = 0.28, interquartile range (IQR) = 0.112] and surface moisture indices (median = 0.14, IQR = 0.079), and the second lowest surface brightness indices (median = 0.11, IQR = 0.017) amongst the BFTs (Fig. 2A–C; Tables S1). Lichens had the second highest chlorophyll (median = 0.21, IQR = 0.076), the highest surface moisture (median = 0.15, IQR = 0.085), and the lowest surface brightness indices (median = 0.10, IQR = 0.026). Darkly pigmented cyanobacteria had chlorophyll (median = 0.15, IQR = 0.037) and surface moisture indices (median = 0.08, IQR = 0.050) that were significantly lower than the mosses and lichens, and surface brightness indices (median = 0.12, IQR = 0.012) that were significantly higher than the mosses and lichens. Finally, lightly pigmented cyanobacteria had the lowest chlorophyll (median = 0.12, IQR = 0.012) and surface moisture indices (median = 0.05, IQR = 0.028), and the highest surface brightness indices (median = 0.16, IQR = 0.015) amongst the BFTs.

At the UAS-scale, where we were able to identify early- and late-successional biocrust communities, we found similar patterns (Fig. 2D–F). Late-successional, darkly pigmented biocrust communities that represented a mixture of mosses, lichen, and darkly pigmented cyanobacteria had significantly higher chlorophyll indices (median = 0.23, IQR = 0.049), significantly lower surface temperature (median = 0.56, IQR = 0.070), and significantly lower surface brightness indices (median = 0.08, IQR = 0.022) relative to biocrust communities made up of early-successional, lightly pigmented cyanobacteria (Table S2). These differences also persisted across experimental treatments.

Significant differences in BFT sensitivities to experimental treatments were also found through additional analysis of isolated spectra collected using a field spectroradiometer (Fig. S3; Tables S3–S6). The cyanobacteria BFTs showed relatively reduced differences in chlorophyll, moisture, and brightness indices across experimental treatments (Fig. S3; Table S3-S4). By contrast, lichens were found to have reduced moisture (median = 0.11, IQR = 0.053) and increased brightness (median = 0.11, IQR = 0.016) indices under the altered precipitation + warmed plots relative to the moisture (median = 0.18, IQR = 0.079) and brightness (median = 0.09, IQR = 0.020) indices measured under control plots (Fig. S3; Table S5). Mosses experienced reduced chlorophyll (median = 0.27, IQR = 0.111) and moisture (median = 0.12, IQR = 0.030) indices under the altered precipitation plots compared to chlorophyll (median = 0.31, IQR = 0.024) and moisture (median = 0.21, IQR = 0.058) indices under control plots (Fig. S3; Table S6). Mosses also experienced reduced chlorophyll (median = 0.20, IQR = 0.101) and moisture (median = 0.11, IQR = 0.083) indices under the warming + altered precipitation plots.

Automated measurements of surface temperature distributed across a subset of plots and active during our sampling period were consistent with the patterns we observed in our thermal remote sensing-based surface temperature index estimates (Fig. S4; Table S7). Control plots were found to have significantly lower surface temperatures (median = 21.8 °C, IQR = 8.65 °C) than the plots that experienced warming (median = 27.2 °C, IQR = 4.38 °C). Notably, these measurements were made when the warming treatment was turned off for multiple days during the campaign and thus were driven by differences in surface energy balance properties including albedo, roughness, and latent heat flux. Automated surface temperature measurements that targeted biocrust functional groups were also consistent with the patterns we observed in our thermal remote sensing-based surface temperature index estimates (Fig. S4; Table S8). Lightly pigmented cyanobacteria were found to have significantly higher surface temperatures (median = 26.7 °C, IQR = 4.90 °C) than darkly pigmented cyanobacteria (median = 16.2 °C, IQR = 5.30 °C) and moss (median = 21.8 °C, IQR = 6.02 °C) biocrust functional types. Lichens are not included here since there were no automated surface temperature measurements that targeted lichens at the experimental site. Automated surface soil moisture measurements made by the same sensors were less conclusive (Fig. S4; Table S7 and S8). We found very low soil surface gravimetric water content across plots, due to the lack of precipitation throughout the weeks leading up to sampling, and no significant differences across treatments nor among biocrust functional types.

### Plot-level indices

We first evaluated our scaling framework by spectrally aggregating our field-scale hyperspectral data to match the UAS and WorldView-3 multispectral bands (see “Methods” section). Comparison across field, UAS, and WorldView-3 band estimates reveals positive relationships confirming the effectiveness of our scaling approach (Fig. S5). WorldView-3 showed a relatively dampened change in reflectance across spectral bands, with relatively high reflectance in the blue bands and relatively low reflectance in the red and near-infrared bands.

**Fig. 3:**
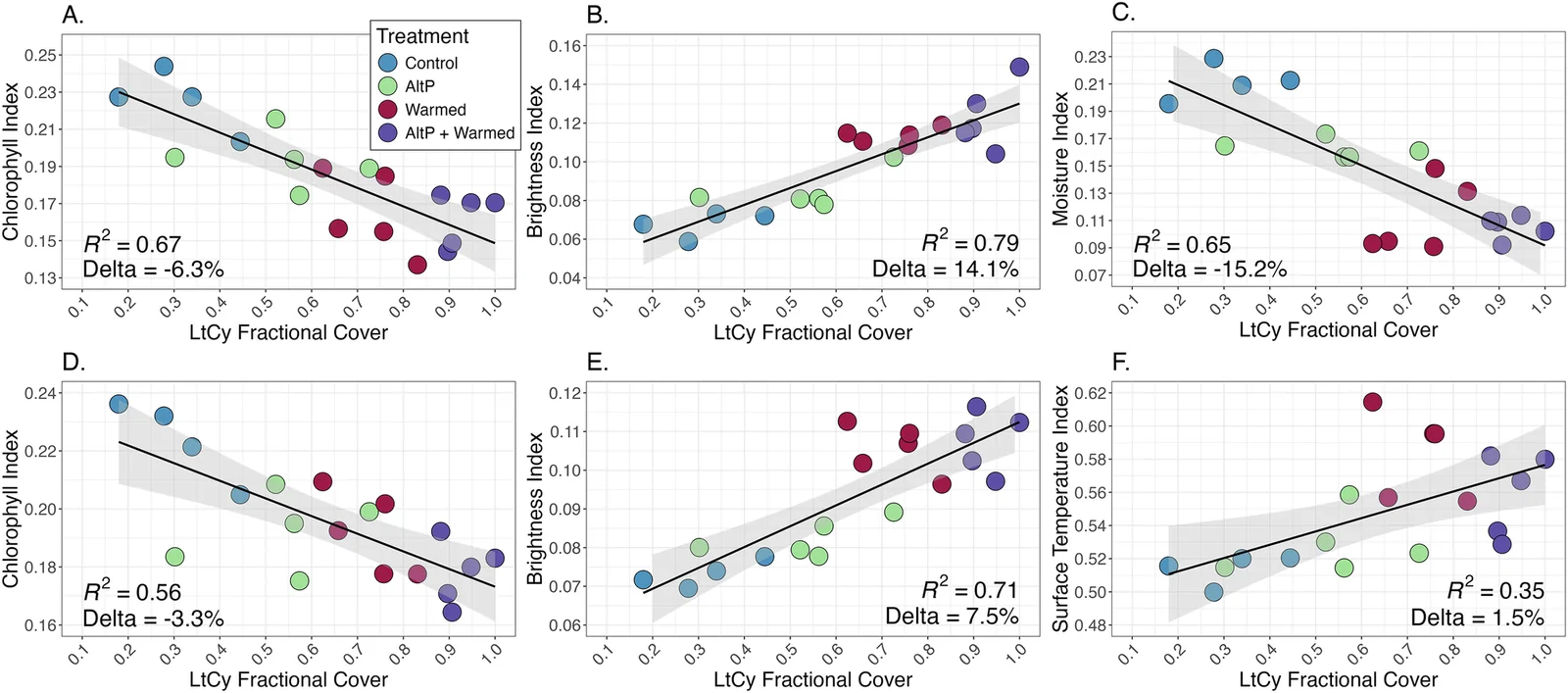
The relationship between remotely sensed functional indices and lightly pigmented cyanobacteria (LtCy) fractional cover across all experimental plots. The top row shows field spectroradiometer estimates of chlorophyll (**A**), brightness (**B**), and moisture (**C**) indices and their relationship with light cyanobacteria fractional cover. The bottom row (**D**–**F**) shows uncrewed aerial system (UAS) estimates of chlorophyll (**D**), brightness (**E**), and temperature (**F**) indices and their relationship with light cyanobacteria fractional cover. Light cyanobacteria fractional cover was estimated as the ratio of light cyanobacteria cover to the sum of total biocrust functional types (BFT) cover (i.e., the sum of light cyanobacteria, dark cyanobacteria, lichen, and moss cover). Each circle in the plot represents a single plot and is colored by treatment: control (Control), altered precipitation (AltP), warming (Warmed), and altered precipitation and warming combined (AltP + Warmed). R^2^ represents the proportion of the variance for a given climate mitigation index that’s explained by lightly pigmented cyanobacteria fractional cover. Delta represents the percentage change for a given climate mitigation index per 0.1 change in light cyanobacteria fractional cover. All relationships (**A**–**F**) are significant at a *p* < 0.0001 level.

Using a combination of plot-scale field- and UAS-based estimates, we found a clear trend in climate mitigation indices: chlorophyll index (field- and UAS-based), surface brightness index (field- and UAS-based), surface moisture index (field-based only), and surface temperature index (UAS-based only) as largely moss fractional coverage decreased and light cyanobacteria fractional coverage increased in the warmed and altered precipitation and warmed plots (Fig. 3). Lightly pigmented cyanobacteria relative to total BFT fractional cover explained 67% and 56% of the variance in the chlorophyll index, which was found to significantly decrease by 6.3% and 3.3% for every 10% change in the relative proportion of lightly pigmented cyanobacteria fractional cover for the field spectroradiometer- and UAS-based estimates, respectively (Fig. 3A, D). Lightly pigmented cyanobacteria relative to total BFT fractional cover explained 79% and 71% of the variance in the brightness index, which was found to significantly increase by 14.1% and 7.5% for every 10% change in the relative proportion of lightly pigmented cyanobacteria fractional cover for the field spectroradiometer- and UAS-based estimates, respectively (Fig. 3B, E). Lightly pigmented cyanobacteria fractional cover explained 65% of the variance in the moisture index, which was found to significantly decrease by 15.2% for every 10% change in the relative proportion of lightly pigmented cyanobacteria fractional cover for the field spectroradiometer-based estimates (Fig. 3C). Lightly pigmented cyanobacteria fractional cover explained 35% of the variance in the temperature index, which was found to significantly increase by 1.5% or 0.3 °C for every 10% change in the relative proportion of lightly pigmented cyanobacteria fractional cover in the UAS-based estimates (Fig. 3F, Fig. S6). In all cases, the relationship between the relative proportion of lightly pigmented cyanobacteria fractional cover and climate mitigation index was statistically significant (*p* < 0.001).

Repeating this analysis with a focus on vascular plant fractional cover, we found a relatively minor role of vascular plants in driving changes in most components of climate mitigation potential across treatments (Fig. S7). Vascular plant fractional cover was found to have a substantial impact on plot chlorophyll and explained 42% and 61% of the variance in the chlorophyll index for the field spectroradiometer- and UAS-based estimates, respectively (Fig. S7A, D). By contrast, vascular plant fractional cover was not a significant factor in explaining plot-level differences in surface brightness (Fig. S7B, E), surface moisture (Fig. S7C), or surface temperature (Fig. S7F).

**Fig. 4:**
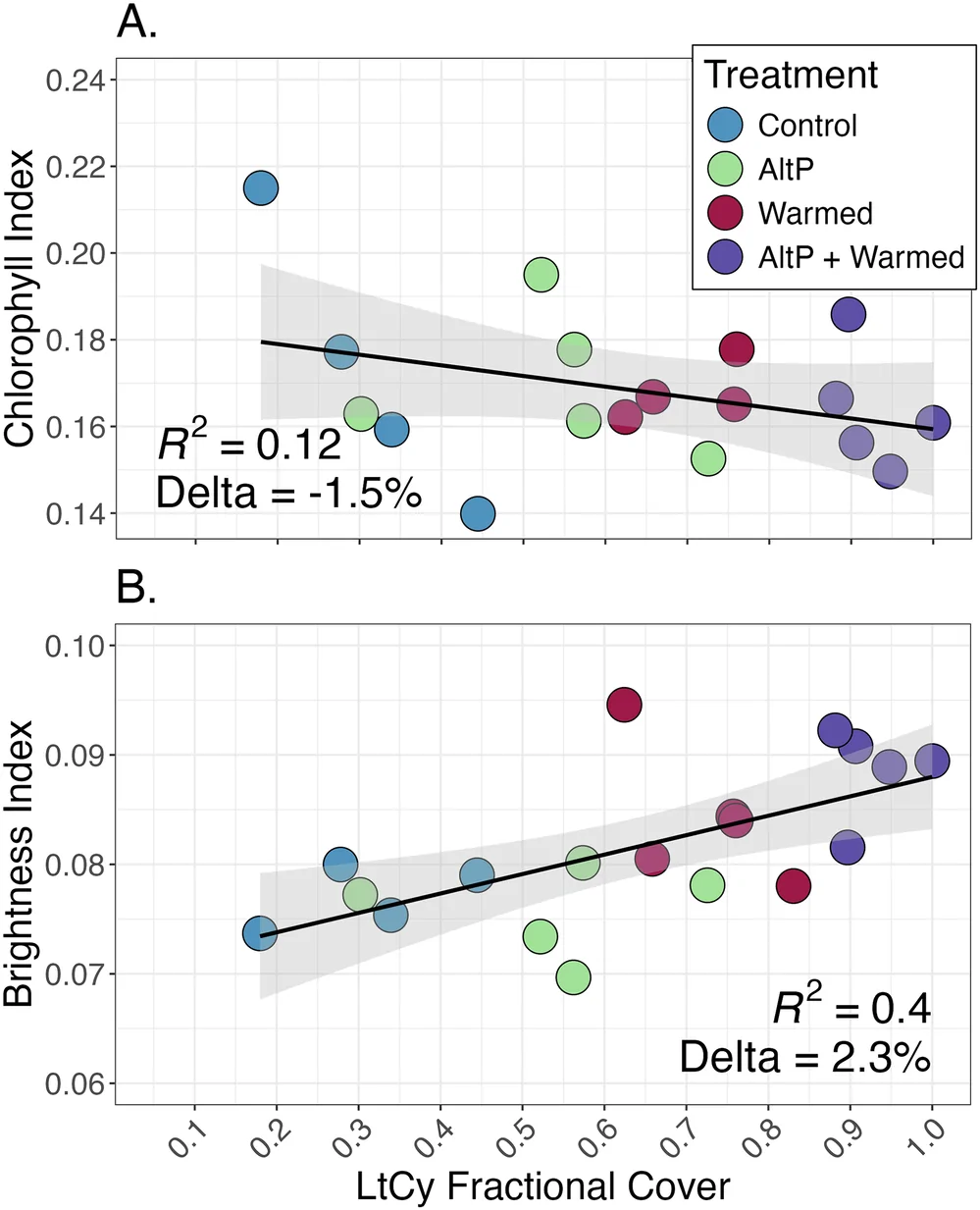
The relationship between WorldView-3 functional indices and light cyanobacteria (LtCy) fractional cover across all experimental plots. Satellite observations of chlorophyll (**A**) and brightness (**B**) indices and their relationship with light cyanobacteria fractional cover. Satellite observations used to derive these indices were from a WorldView-3 scene that was collected during the field campaign. Light cyanobacteria fractional cover was estimated as the ratio of light cyanobacteria cover to the sum of total biocrust functional types (BFT) cover (i.e., the sum of light cyanobacteria, dark cyanobacteria, lichen, and moss cover). Each circle in the plot represents a single plot and is colored by treatment: control (Control), altered precipitation (AltP), warming (Warmed), and altered precipitation and warming combined (AltP + Warmed). *R*^2^ represents the proportion of the variance for a given climate mitigation index that’s explained by light cyanobacteria fractional cover. Delta represents the percentage change for a given climate mitigation index per 0.1 change in light cyanobacteria fractional cover.

Plot-scale WorldView-3 estimates of chlorophyll and brightness index were consistent with the field- and UAS-based estimated although the slopes with the relative proportion of lightly pigmented cyanobacteria cover were notably reduced (Fig. 4). Satellite-based chlorophyll index was found to decrease by 1.5% for every 10% change in the relative proportion of lightly pigmented cyanobacteria fractional cover (Fig. 4A). However, the relative proportion of lightly pigmented cyanobacteria fractional cover explained only 12% of the variance in the satellite-based chlorophyll index and the relationship was non-significant. Satellite-based brightness index was found to increase by 2.3% for every 10% change in the relative proportion of lightly pigmented cyanobacteria fractional cover (Fig. 4B). The relative proportion of light cyanobacteria fractional cover explained 40% of the variance in the satellite-based brightness index, and the relationship was statistically significant (*p* < 0.001).

## Discussion

### Biocrust climate mitigation potential

We found clear differences in climate mitigation potential across biocrust functional types (Fig. 2). In particular, late-successional mosses had the highest climate mitigation potential due to their notably high chlorophyll (median = 0.28; IQR = 0.112) and moisture indices (median = 0.14, IQR = 0.079) and low brightness (median = 0.11; IQR = 0.017) and surface temperature indices (median = 0.56, IQR = 0.070). By contrast, early-successional, lightly pigmented cyanobacteria had the lowest climate mitigation potential due to their notably low chlorophyll (median = 0.12, IQR = 0.012) and moisture indices (median = 0.05, IQR = 0.028) and high brightness (median = 0.14, IQR = 0.026) and surface temperature indices (median = 0.64, IQR = 0.040). Surface temperature measurements recorded across select plots also indicated that lightly pigmented cyanobacteria had significantly higher surface temperatures (median = 26.7 °C, IQR = 4.90 °C) than dark cyanobacteria (median = 16.2 °C, IQR = 5.30 °C) and moss (median = 21.8 °C, IQR = 6.02 °C). This result can seem counterintuitive, as it could be predicted that the higher reflectance of lightly pigmented cyanobacteria-dominated soils could mean higher albedo^18^ and thus cooler soil temperatures^38^, as more energy is reflected back to the atmosphere. The data we collected indicate more complicated controls. In particular, the cooler soil temperatures were associated with wetter soils, with connections to the effects different biocrust communities have on dryland hydrology^15,39,40^.

Our findings are in support of previous research that has found mosses have the highest rates of photosynthesis^41,42^ and surface moisture^15^ and the lowest surface temperatures^15^ relative to other BFTs. Chen et al. (2019)^40^ provided evidence that the higher surface area-to-volume ratio of mosses resulted in greater rates of surface evaporation, which could explain the relatively high surface moisture and reduced surface temperature indices that we found in our study. Further, the structural changes to biocrust communities, both with composition shifts and with changes to the biocrust ‘bumpiness’, which is small-scale topography characteristic of biocrusts in systems with freeze-thaw dynamics, could also drive substantial changes in surface energy balance^18^. Further work could benefit our understanding of the mechanisms driving these patterns. Nevertheless, the finding that later successional biocrust communities can cool dryland soils underscores the importance and multifaceted nature of biocrust controls over ecosystem function and future climate. Notably, these controls and patterns could vary across not only space, but also time. The soils were relatively dry during our measurement period, which is their most common state, yet differences in the control of climate mitigation indices at wetter times are possible and even likely, showcasing the importance of further assessment of biocrusts’ climate mitigation controls. Overall, the large and consistent differences in functional indices across BFTs indicate that any shifts in biocrust community composition have the potential to drive large-scale changes in ecosystem climate mitigation potential across biocrust-dominated drylands.

### Biocrust degradation with climate change

Major shifts from a late-successional, darkly pigmented to an early-successional, lightly pigmented biocrust community were observed across warmed and warmed + altered precipitation plots. For instance, in the warmed + altered precipitation treatment, fractional coverage of late-successional mosses decreased by an average of ~27% whereas the fractional cover of early-successional, lightly pigmented cyanobacteria increased by an average of ~36%. Moss and to a lesser extent lichen mortality due to warming and/or altered precipitation has been previously explained by a combination of interacting factors including: (i) more rapid desiccation with warming resulting in reduced carbon uptake per rainfall event; (ii) increased respiration rates with warming resulting in an increase in baseline carbon losses; (iii) increase carbon losses with altered precipitation in scenarios when the cost incurred to initiate metabolic activity at the onset of hydration is greater than the carbon gains from photosynthesis^20–22^. Together, these factors drive a carbon deficit that can lead to an increase in the occurrence of mortality, likely by carbon starvation, of the late-successional, darkly pigmented mosses and lichens in the warmed and warmed + altered precipitation treatment plots. This climate change-driven reversal of biocrust succession ultimately resulted in the dominance of early-successional, lightly pigmented cyanobacteria within these plots^20–22^.

We also found clear degradation in the climate mitigation potential of late-successional BFTs in warmed and/or altered precipitation plots. Specifically, late-successional mosses in warmed plots experienced a ~19% reduction in chlorophyll index, ~33% reduction in surface moisture index, and a ~9% increase in surface brightness index compared to control plots. This finding expands on previous research at this study site that focused on warming-driven reductions in moss cover^20,21^, by showing additional degradation due to loss of functional capacity of the mosses that remain in warmed plots. Late-successional mosses in altered precipitation plots also experienced an average ~15% reduction in chlorophyll index compared to control plots. This again expands on previous research at this site that showed high sensitivity of moss cover to altered precipitation by more directly showing moss functional degradation under altered precipitation^20,21^.

Thus, current rates of climate warming and hydroclimate change could be reducing biocrust function prior to any state shift in biocrust community composition. These changes in biocrust function may be currently unaccounted for since they are undetectable by traditional community composition analysis alone. Given well-documented warming and shifts in precipitation timing and intensity across the western U.S.^25^, major climate change-induced shifts in moss functional capacity are likely underway, underscoring the importance of an improved, scalable understanding of how an altered climate might affect biocrusts’ control over feedbacks to further change. This study thus provided a unique opportunity to assess change, as the climate treatments resulted in altered conditions within the same background soil (facilitating direct spectral comparisons) and across two decades, allowing us to explore the system effects of altered climate both within the enduring communities and across communities that have shifted.

### Biocrust degradation climate warming amplification feedback

The large shift in biocrust community composition toward early-successional community cover resulted in major degradation of plot-level functional capacity (Fig. 3, Fig. S6). Plot-level photosynthetic capacity, surface moisture, surface brightness, and surface temperature were changed by −6.3%, −15.2%, +14.1%, and +1.5% or 0.3 °C for every 10% increase in lightly pigmented cyanobacteria cover relative to total biocrust cover, respectively. We further show that while vascular plant cover varied substantially across plots, it explained very little of the variance in functional traits compared to the biocrusts (Fig. S7).

Our findings are consistent with previous research that found moss cover had a surface albedo 43.4% lower than that of the bare soil, due to its increased darkness (43.7%), roughness (90.4%), and increased moisture (20.7%)^15,18^. In particular, Xiao and Bowker (2020) found that the increased energy absorption by the moss cover did not consistently increase soil temperature as might be expected; instead, soil temperature increased by up to 9.3 °C under dry conditions but decreased by as much as 11.4 °C under wet conditions, resulting in a net cooling over a 2-year study period. Another independent study focused on the tundra similarly found that lichen cover was a major factor driving reductions in surface temperatures despite their dark appearance, indicating the findings presented here may extend to tundra ecosystems^43^. Multiple larger-scale studies underscore the importance of our findings by confirming that shifts in biocrust community and the associated changes in surface moisture and albedo impact surface energy balance at the landscape and regional scale^39,44,45^.

In opposition to our findings, Couradeau et al.^38^ found that increases in late-successional, darkly pigmented cyanobacteria cover increased soil temperatures by as much as 10 °C. This discrepancy could be due to multiple factors and may highlight the complementarity of the studies’ results: (1) our study included mosses and lichens, which were found to have much higher climate mitigation potential compared to late-successional dark cyanobacteria; (2) our measurements were made in the field and thus factors like surface evaporation, surface roughness, and residual soil moisture were maintained; and (3) Couradeau et al.^38^ transplanted their biocrusts from a cold (Colorado Plateau) to a hot desert (Sonoran Desert) environment, which may have affected the balance between the physical and biological processes that impact surface energy balance (e.g., potential for reduced biocrust surface area and roughness). Thus, Couradeau et al.^38^ focused on the potential for biocrust pigmentation to have large effects on soil temperature, which complements our findings of multifaceted, bidirectional in situ controls. Additional research that explores the importance of these separate but interacting factors, as well as seasonal and interannual changes in biocrust functional activity, could be a powerful future research priority.

**Fig. 5:**
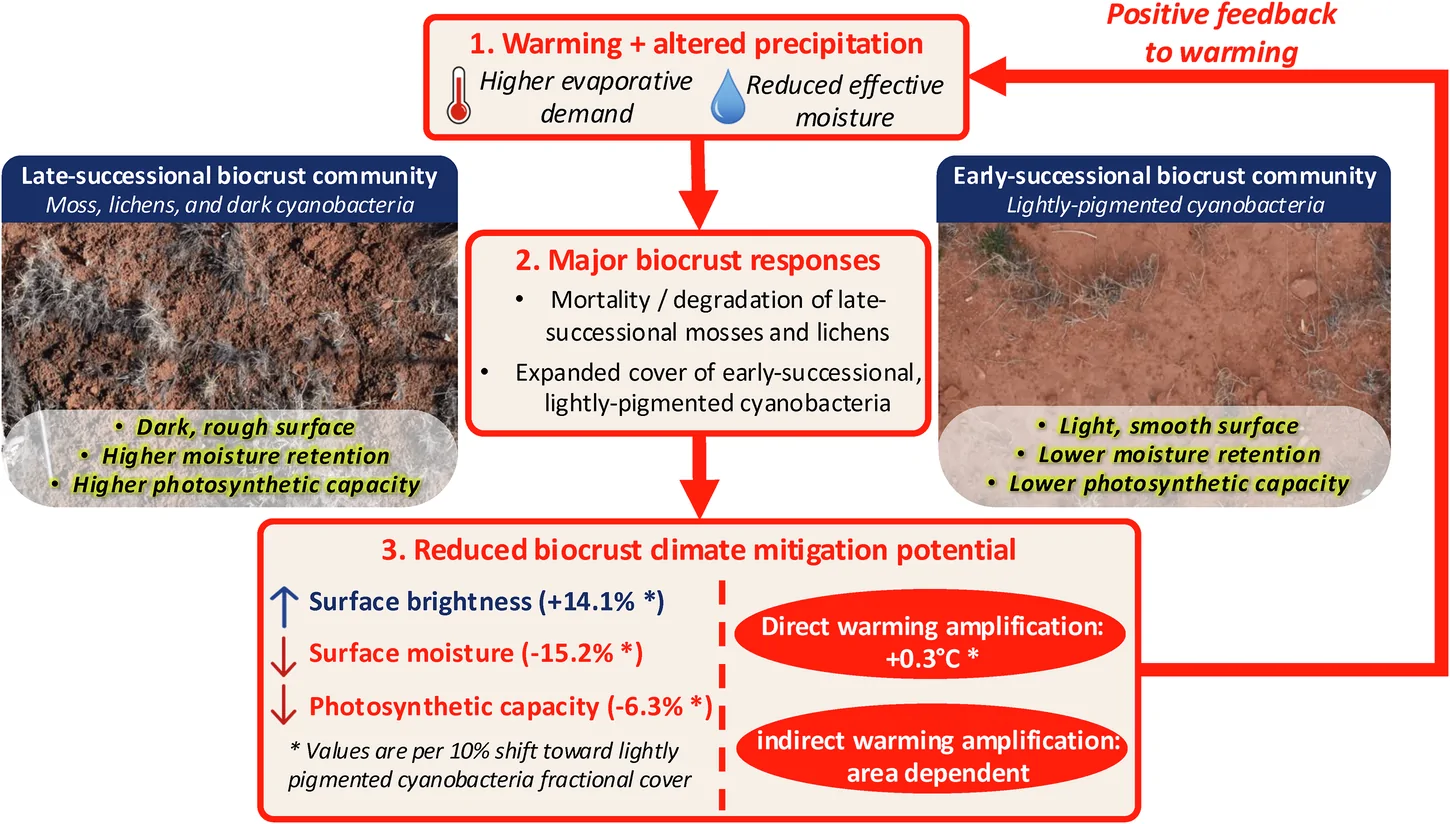
Conceptual figure illustrating a potential biocrust degradation climate warming amplification feedback. Warming + altered precipitation leads to moss and lichen degradation/mortality, most likely due to carbon starvation. Loss of these darkly pigmented, late-successional biocrusts leads to the dominance of early-successional, lightly pigmented cyanobacteria coverage. The net effect of this biocrust community composition shift is a substantial reduction in climate mitigation potential and both a direct and indirect warming amplification feedback.

Ultimately, the substantial reduction in biocrust contributions to ecosystem function and climate mediation with warming and warming + altered precipitation that we have identified here portends a novel and likely already occurring biocrust degradation – climate warming amplification feedback (Fig. 5). The warmer surface temperature of the lightly pigmented compared to the darkly pigmented biocrust communities, despite the higher albedo of the former, implies that increased sensible heat flux of the lightly pigmented cyanobacteria more than compensate for their higher albedo. We find an additional surface warming of ~0.3 °C for only a 10% shift in biocrust community composition – a shift that’s likely already occurring across the 12% of the terrestrial land surface covered by biocrusts^46^. Additionally, reductions in photosynthetic capacity associated with the conversion from darkly pigmented mosses to lightly pigmented cyanobacteria indicate significant ecosystem functional degradation and additional climate warming through reduced uptake of atmospheric CO_2_ over space and time. Previous estimates indicate biocrusts account for roughly 0.58 Pg C per year of global gross primary productivity^46^, which translates to a net global cooling potential of roughly ~0.08 °C by the end of the century^47^. Our findings indicate that this indirect cooling pathway of biocrusts could be reduced by up to 66% with a large-scale shift toward lightly pigmented cyanobacteria communities. Overall, given the clear dominance of warming amplification feedbacks found here, it is likely that the net result of these direct (surface temperature) and indirect (carbon uptake) climate mitigation pathways may be net warming, triggering continued ecosystem functional degradation and potential warming amplification.

Our findings provide a nuanced update to previous efforts that focused only on albedo by providing a more comprehensive view of the climate mitigation benefits provided by late-successional biocrust communities^18,38^. While our study represents a snapshot in time, previous efforts confirmed that direct changes in albedo (local surface warming) were overcompensated by changes in surface moisture and roughness (local surface cooling) for moss cover compared to bare ground, resulting in a net cooling over a 2-year study period^15^. Further, in factoring in the climate mitigation provided by mosses, quantified here as photosynthetic potential, which has been previously found to be a reliable indicator of moss carbon fixation rates^41^, it becomes even clearer that the loss of mosses and more generally late-successional biocrust communities with climate change could result in major biocrust function degradation and a net warming amplification feedback^27^. The acute sensitivity of particularly mosses to both altered precipitation and warming indicates that significant increases in climate warming amplification processes are likely already occurring at the regional scale.

### Biocrust monitoring across spatiotemporal scales

Monitoring of biocrust community composition and function across spatial and temporal scales over large land areas could improve our understanding of their substantial but unquantified contribution to dryland function, including climate mitigation^8,10,26^, exceptional sensitivity to climate change^20,21^, and the rapid changes in climate already occurring across the western U.S.^24,25^. As a proof-of-concept, we explored the potential to estimate plot-level chlorophyll and brightness index from very high spatial resolution satellite observations from WorldView-3 (1.24 m) (Fig. 4). Notably, as we scaled from field-, to UAS-, to satellite-based estimates, the magnitude of the change in each climate mitigation trait was reduced. For instance, the change in surface brightness was estimated to be +14.1%, +7.5%, and +2.3% for field-, UAS-, and satellite-based estimates, respectively (Figs. 3, 4). This is likely due to increasing edge effects from untreated pixels as the spectral and spatial resolution of the measurements became coarser from the field to UAS to satellite scale (Fig. S5). Additionally, uncorrected atmospheric issues could be increasingly contributing to uncertainty or introducing bias from the field to UAS to satellite scale. Chlorophyll indices experienced even larger signal reduction: −6.3%, −3.3%, −1.5% at the field-, UAS-, and satellite scale, respectively. Chlorophyll indices are likely further biased by Raleigh scattering, which has a larger impact on visible compared to near-infrared wavelengths and increases in importance with atmospheric path length from UAS to satellite measurements (Fig. S5). Nevertheless, the consistent pattern of reduced photosynthetic potential and increased surface brightness from field to satellite measurements demonstrates the emerging potential to monitor biocrust community composition and function at high spatiotemporal resolution across global dryland regions by leveraging next-generation spaceborne platforms. Fusion of high spatial resolution observations (e.g., WorldView-3, PlanetScope) that can capture the intricate spatial heterogeneity of biocrusts, with high spectral resolution observations (e.g., NASA EMIT, ESA EnMAP, Landsat Next) that can capture multiple functional traits and climate mitigation indices offers a potential way forward^30,48^. Large-scale biocrust monitoring efforts to quantify and build resilience in existing biocrust communities could provide innovative options for sustaining these organisms and reducing climate feedbacks, and remote sensing approaches offer under-utilized tools for vastly improving our capacity to assess, model, and make decisions for these foundational soil communities.

### Enhancement of global change experiments through the integration of remote sensing

We demonstrate an approach in which we integrate multiscale remote sensing measurements (field, UAS, and satellite) within a long-term global change experiment to reveal a novel biocrust degradation – climate warming amplification feedback that is likely already occurring across vast global dryland ecosystems. Further, our approach enabled a nuanced enhancement to previous efforts, which by focusing only on albedo, came to an opposite conclusion – expansion of early-successional light cyanobacteria and the loss of late-successional mosses could result in surface cooling due to albedo increases^18^. Our more comprehensive view was achievable by the integration of multiscale remote sensing measurements over the optical and thermal regions of the electromagnetic spectrum, which revealed substantial increases in surface temperature in climate change treatment plots, despite major increases in light cyanobacteria fractional coverage and corresponding increases in surface brightness (Figs. 3, 5). These findings underscore the importance of monitoring, restoring, and protecting late-successional biocrusts across global drylands for their substantial role in ecosystem function and climate mitigation. The approach developed here could be easily adapted for application in a wide range of existing long-term global change experiments to complement traditional methods and extend the inferences gained from these time-, labor-intensive, and expensive undertakings^49^.

## Methods

### Study site

The study site is located near Castle Valley, Utah (38.67° N, 109.42° W) on the Colorado Plateau (Fig. 1), a semiarid region of primarily grasslands and shrublands in which biocrusts constitute up to 70% of the living soil cover and are a substantial part of the ecosystem^50^. The site is characterized by a mean annual temperature range of 4 to 13 °C and a mean annual precipitation range of 205–510 mm. The soils of the study site are classified as sandy loam, calcareous, Rizno series^51^. Biocrust community composition at the site is made up of four major assemblages, which we grouped into major BFTs: lightly pigmented cyanobacteria (*Microcoleus vaginatus*), darkly pigmented cyanobacteria (*Nostoc* spp.*, Scytonema* spp., and *Tolypothrix* spp.), mosses (*Syntrichia caninervis*), and lichens (*Collema tenax* and *C. coccophorum*). Vascular plants at the site include the shrub *Atriplex confertifolia*, the perennial grass species *Achnatherum hymenoides* and *Pleuraphis jamesii*, and the exotic annual grass *Bromus tectorum*.

The study site has 20 plots, each measuring 2 × 2.5 m in a randomized block design to account for downslope spatial variation across the site. The plots were originally distributed into four treatments with five replicates each: control (ambient conditions), warming, altered precipitation, and a combination of warming with altered precipitation. Infrared heat lamps were mounted approximately 1.3–1.5 m above the soil surface to increase soil surface temperatures above ambient conditions. The warming treatment began in October 2005 and raised soil surface temperatures by 2 °C above ambient. In June 2008, the warming treatment was adjusted to 4 °C above ambient to better align with updated climate projections for the region^50,52,53^. Southeast Utah, the location of the research site, has already experienced relatively high-end warming, with updated projections of 5 °C warming by 2100^54^. In addition to the warming treatment, background variations in temperature continue to provide realistic year-to-year variation in temperature. Inactive “dummy” lamps were also installed above control and watered plots to standardize for any unintended effects of lamp infrastructure. The altered precipitation treatment was designed based on output from climate models that forecasted climate change may drive a shift in hydroclimate toward that of hotter North American deserts, which experience more frequent summer rainfall^55–57^. The altered precipitation treatment occurred for six summers (2006–2012) and ended due to rapid and sustained shifts in the composition of biological soil crust communities, particularly the loss of late-successional moss biocrust communities^21^. Since 2012, the watered plots have been recovering from the precipitation effects^20^.

### Plot measurements

Biological soil crust communities were assessed for each plot prior to the field campaign using 1 × 1 m frames and a point intercept method with 100 points in each frame. Point frames were read in each of the four quadrants of each plot, resulting in an accurate representation of fractional cover at the whole-plot scale. Biocrusts were recorded at the functional-group level and included lightly pigmented cyanobacteria, darkly pigmented cyanobacteria, lichen, and mosses. Vascular plants were recorded at the species level and were then aggregated into an estimate of total cover for this analysis. We estimated plot-level biocrust functional type and vascular plant cover by dividing the number of readings for each cover type by the total readings per plot. We further assessed the quality of the fractional cover estimates for each plot through visual inspection of high-resolution photos. As a result, fractional cover estimates of one of the control plots were flagged and removed from all plot-level analyses. We note that biocrusts found under vascular plant canopies were counted in the point intercept-based estimates of fractional cover. This introduced an unavoidable source of uncertainty in our comparison of plot-level biocrust fractional cover and remote sensing-based climate mitigation potential estimates. Further details on the method used for estimating plot-level fractional cover can be found in ref. ^21^.

Direct biocrust surface soil moisture and temperature measurements were collected across a subset of the experimental plots and were used in this analysis for evaluation of our remote sensing indices. The sensors were designed to measure both temperature and moisture integrated across a 5 mm depth from the soil surface^58,59^. One soil sensor per plot was placed in the interspace between plants within the biocrust type that was the most dominant cover in the plot. Measurement records were quality filtered prior to data analysis. Soil moisture data were collected in Siemens values, and any data that were collected when soil temperatures read below zero were converted to zeros, and all negative Siemens values were changed to zero. The cleaned soil moisture data were converted from Siemens into gravimetric water content using a conversion factor of 0.01594, which was determined from calibration measurements on soils from the site as described in Howell et al.^58^.

### Remote sensing field campaign

We conducted a remote sensing field campaign during a time of year when biocrusts have the potential to be physiologically active at this site, from February 12–14, 2022^7,16,60^. The warming treatment was paused during the campaign, and the warming treatment infrastructure was removed the day before the start of the field campaign so that the warming treatment and infrastructure did not directly affect the remote sensing measurements. Finally, the last recorded precipitation event (2.1 mm of rain and 1.68 cm of snow) occurred 22 days before sampling, and thus our remote sensing measurements were not influenced by a short-term precipitation response. Due to this substantial time since last precipitation, at the time of measurement the site’s soils were relatively dry (Fig. S4).

As part of this campaign, we collected ground-based hyperspectral data at the BFT and plot level using a handheld spectroradiometer. We also collected uncrewed aerial system (UAS) natural color red-green-blue (RGB), 10-band multispectral, and thermal infrared data at the BFT and plot scale. We note that due to the relatively coarse scale of the aerial UAS collection, BFT were grouped more broadly into early-successional (light cyanobacteria) and late-successional (dark cyanobacteria, lichens, and mosses) functional types. We additionally incorporated an 8-band multispectral WorldView-3 image from February 12, 2022, to explore the potential of monitoring treatment effects at the plot scale using very high-resolution (1.24 m) satellite data. The warming treatment infrastructure was removed during the WorldView-3 overpass so that plot infrastructure did not directly affect the satellite, which had a clear view of all study plots and was unimpacted by plot infrastructure. The experimental site was ideally suited for the incorporation of multiscale remote sensing techniques given the uniformity of soil type and texture across plots and the overall size and location of the plots within the site. We describe these collected datasets in more detail below.

### Field spectral data and processing

Field-level spectral data were collected February 13–14, 2022. We measured BFT- and plot-level hyperspectral reflectance within the wavelength range of 350 to 2500 nm at a 1 nm spectral resolution using an Analytical Spectral Devices (ASD) FieldSpec3 spectroradiometer (hereafter referred to as “field spectroradiometer”, Malvern Panalytical, Worcestershire, UK). BFT-level reflectance measurements were made using a field Contact Probe assembly^61^. The Contact Probe is designed for contact measurements and has a diameter of approximately 25-mm and uses an internal halogen bulb light source with a color temperature of 2900 K. When collecting spectra at the BFT level, for each of the 20 plots, isolated BFTs (i.e., lightly pigmented cyanobacteria, darkly pigmented cyanobacteria, lichen, and moss) were identified and then non-destructively sampled by holding the contact probe directly against the sample. We slightly altered the position of the Contact Probe and collected five spectra from each sample to account for small differences in the sample coverage and sample-sensor geometry. Plot-level reflectance measurements were made using an ASD Pistol Grips assembly. The ASD Pistol Grips has a 15-degree field of view and is designed for bare-fiber measurements at a fixed height above a target sample using the sun as a light source. All measurements were made in full sun conditions and were not impacted by cloud cover. When collecting spectra at the plot-level, for each of the 20 plots, we collected a total of 15 equally spaced spectra per plot to obtain full coverage with adequate overlap between measurements. We slightly altered the position of the Pistol Grips and collected five spectra from each sampling location to account for small differences in the coverage and sample-sensor geometry. Finally, for each plot, we averaged all spectra across the 15 sampling locations to estimate plot-level spectral reflectance.

For all collected spectra, we used a Spectralon white reference panel to convert digital number values into estimates of reflectance. We strictly followed the instrument operation procedures frequently (Malvern Panalytical, 2014), including turning on the spectroradiometer at least 30 min before collecting any spectra and optimizing the spectroradiometer and checking the white reference every 5 min or whenever spectra exhibited abrupt discontinuities. All collected spectra were saved as binary files, which were imported into R using the “asdreader” package^62^. We excluded the reflectance within 350–400 nm and 2450–2500 nm from the following analyses due to the noise within these wavelength ranges as reported in previous studies^61^.

### UAS spectral data and processing

Uncrewed aerial system (UAS) data were collected February 12–14, 2022, in collaboration with the U.S. Geological Survey (USGS) National Uncrewed Systems Office^63^. A series of sensors described in the following paragraphs were flown on a Matrice 600 Pro (SZ DJI Technology Co., Ltd., Shenzhen, China) hexacopter UAS. Flights were conducted using terrain following in the IGNIS App (Drone Amplified, Lincoln, NE, USA) to maintain a flight altitude of 31 m (100 ft) above ground level, capturing high-resolution aerial imagery across the study area. Survey control was established using 10 AeroPoint smart targets (Propeller Aero, Sydney, Australia) as temporary ground control points (GCPs) distributed throughout the survey area each day. GCPs were post-processed using the Propeller Corrections Network, which integrates base stations worldwide to correct each GCP with satellite observations from an existing nearby base station. Using this protocol, the average horizontal and vertical global positional accuracy associated with each GCP was calculated to be approximately 40 mm and 50 mm, respectively. All UAS data were processed using the photogrammetry software package Agisoft Metashape Professional^64^. The processing workflow consisted of aligning the images, georeferencing the data by importing and refining GCP locations, iteratively filtering out tie points with high errors, and exporting structure-from-motion data products including digital surface models and orthomosaics. Additional details and processing steps are described below for each sensor type.

A Ricoh GRII (Ricoh Imaging Company, Ltd., Tokyo, Japan) camera was used to collect high spatial resolution RGB imagery for the full study site on February 12, 2022 (Fig. 1). The RGB images were processed to generate a 3-band RGB orthomosaic with a ground resolution of ~0.8 cm per pixel.

A MicaSense RedEdge-MX Dual (AgEagle Aerial Systems Inc., Wichita, KS, USA) was used to collect 10-band multispectral imagery across the visible and near-infrared wavelengths on February 13, 2022, for the full study site (Fig. 1; Fig. S1). The center wavelength information for each of the 10 bands of the MicaSense Dual System are as follows: 1 - Coastal blue (444 nm), 2 - blue (475 nm), 3 - green (531 nm), 4 - green (560 nm), 5 - red (650 nm), 6 - red (668 nm), 7 - red edge (705 nm), 8 - red edge (717 nm), 9 - red edge (740 nm), and 10 - NIR (842 nm). A calibrated reflectance panel (Panel serial number: RP05-2005276-OB) was imaged on the ground before and after the flight to enable reflectance calibration in Agisoft Metashape software according to MicaSense recommendations. The 16-bit orthomosaic bands were normalized to reflectance values ranging from 0 to 1.0 by dividing by a scale factor of 32,768 using the Metashape Raster Calculator. The final output was a 10-band multispectral reflectance orthomosaic with a spatial resolution of 1.5 cm per pixel. A 3 × 3 m fabric tarp with three gray levels was present in the flight area to externally radiometrically validate the UAS multispectral data. The mean reflectance values across all 10 bands within the dark tarp (with a reflectance level of approximately 0.11), gray tarp (0.30), and white tarp (0.56) regions ranged between 0.10 and 0.12, 0.29 and 0.32, and 0.49 and 0.62, respectively. Plot-level spectral reflectance was estimated by averaging all pixels within a given plot for each spectral band.

A Zenmuse XT2 (SZ DJI Technology Co., Ltd., Shenzhen, China) was used to collect thermal imagery for the full study site on February 13, 2022 (Fig. 1; Fig. S1). The thermal longwave infrared band had a wavelength range from 7.5 to 13.5 μm. Four flights were conducted to capture diurnal changes in surface temperature at 10:33, 12:47, 13:58, and 15:06 local times of day (Fig. S1). The thermal images were processed to generate structure-from-motion orthomosaics with relative, uncalibrated surface temperature observations across the study site at a ground resolution of ~3.0 cm per pixel. Absolute surface temperatures were estimated using the relationship between relative and absolute temperatures from a calibrated thermal flight (Fig. S6). Plot-level relative and absolute surface temperatures were estimated by averaging all pixels within a given plot for each of the thermal flights.

### Satellite spectral data and processing

We also utilized a WorldView-3 (Maxar/Vantor Inc.) image of the study site collected on February 12, 2022, specifically tasked to coincide with our UAS image collections. The multispectral image has a spatial resolution of 0.31 m for the panchromatic band and 1.24 m for eight spectral bands: coastal (397–454 nm), blue (445–517 nm), green (507–586 nm), yellow (580–629 nm), red (626–696 nm), red edge (698–749 nm), NIR1(765–899 nm) and NIR2(857–1039 nm). The radiometrically corrected image pixel values were then converted to spectral surface reflectance using the provided metadata and conversion equations (Updike and Comp)^65^. Experimental plots were manually identified, and then all pixels within a given plot were aggregated to estimate plot-level median surface reflectance across all available bands.

### Plot-level comparison across spectral measurements

We compared spectral surface reflectance estimates across products by aggregating the field spectroradiometer, UAS multispectral data, and satellite WorldView-3 data to the plot level and across shared spectral bands. Field spectroradiometer plot-level median surface reflectance was aggregated using wavelength centroids and bandwidth to match the UAS multispectral camera (10 bands) and the WorldView-3 (8 bands) spectral bands. UAS multispectral and satellite WorldView-3 spectral data were compared across eight overlapping spectral bands that had similar wavelength centroids. We note that this relatively simple approach was used to evaluate whether consistent plot-level trends were detectable across commonly utilized platforms despite known uncertainties introduced due to differences in spectral response functions, bandwidth, radiometric properties, and spatial scale (Fig. S5).

### Functional indices

We derived from the field spectroradiometer- and UAS-based remote sensing observations key functional indices: chlorophyll index, surface moisture index, surface temperature index, and surface brightness index. The chlorophyll index was estimated from hyperspectral and multispectral data using the below equation, which has been previously found to capture variation chlorophyll content in vascular plants and biocrusts^42^:

$${Chlorophyll}\,{Index}=\frac{{NIR}-R}{{NIR}+R}$$

Where, at the field spectroradiometer-scale, NIR represents near-infrared reflectance at 850 nm and R represents red reflectance at 650 nm. At the UAS-scale, NIR was represented by the NIR842 band centered at 842 nm and with a bandwidth of 42 nm and R was represented by the Red650 band centered at 650 nm and with a bandwidth of 16 nm.

The moisture index was calculated from hyperspectral data only using the below equation:

$${Moisture}\,{Index}=\frac{{SWIR}1-{SWIR}2}{{SWIR}1+{SWIR}2}$$

Where SWIR1 represents shortwave infrared (SWIR) reflectance at 1850 nm, and SWIR2 represents reflectance at 1925 nm. Moisture index could only be derived from measurements made by the field spectroradiometer since this was the only instrument with coverage into the SWIR.

The brightness index was calculated from hyperspectral and multispectral data using the below equation:

$${Brightness}\,{Index}=\frac{\sqrt{({G}^{2}+{R}^{2}+{{NIR}}^{2})}}{3}$$

Where, at the field spectroradiometer-scale, G represents green reflectance at 560 nm, and R represents red reflectance at 650 nm, and NIR represents near-infrared reflectance at 850 nm. At the UAS-scale, G was represented by the Green560 band centered at 560 nm and with a bandwidth of 27 nm, R was represented by the Red650 band centered at 650 nm and with a bandwidth of 16 nm, and NIR was represented by the NIR842 band centered at 842 nm and with a bandwidth of 42 nm.

Finally, the temperature index was calculated from relative surface temperature observations that were then normalized by the minimum and maximum temperatures measured across all plots and for all flight times (Fig. S1).

### Conversion factors and statistical analysis

The potential warming associated with loss of biocrust photosynthesis potential reported here was estimated using a standard conversion factor of 0.00165 °C per 1 Pg C^52^. All reported statistical comparisons of distributions were carried out using a Kruskal-Wallis test, followed by post-hoc pairwise comparisons using the Wilcoxon signed-rank test. Linear regression models were utilized for all reported coefficients of determination (*R*^2^) values. All statistical analyses were carried out using R v.4.4.2^66^.

## Supplementary information

Supplementary Information

Transparent Peer Review file

## Data Availability

All data in this paper are publicly available. The field-level data are available via GitHub at https://github.com/wkolby/CastleValley_Campaign_Biocrust_Analysis. The UAS- and satellite-level data are available at https://doi.org/10.5066/P14QORRX^63^.

## Code availability

The code that supports the main findings in this study is available via GitHub at https://github.com/wkolby/CastleValley_Campaign_Biocrust_Analysis.
