# Supplementary material for: Experimental evidence of a biological soil crust degradation climate warming amplification feedback
Source: Commun Earth Environ. 2026 Sep 12;7(1):775. doi: 10.1038/s43247-026-03874-5 (PMC13593566; doi:10.1038/s43247-026-03874-5)
Supplement: Supplementary file 1 [file 43247_2026_3874_MOESM1_ESM.pdf]

Supplementary Information for "Experimental evidence of a biological soil crust degradation climate warming amplification feedback"

William K. Smith[1*], Miguel L. Villarreal[2], Cara Lauria[3], Willliam A. Rutherford[1,4], Stefanie Herrmann[5], Victoria Scholl[6], Armin Howell[3], Mostafa Javadian[1], Fujiang Ji[1], Fangyue Zhang[1], Matthew A. Burgess[6], Raymond Kokaly[7], Benjamin Poulter[8], Sasha C. Reed[3]

[1]School of Natural Resources and the Environment, University of Arizona, Tucson, AZ, USA
[2]U.S. Geological Survey, Western Geographic Science Center, Moffett Field, CA, USA
[3]U.S. Geological Survey, Southwest Biological Science Center, Moab, UT, USA
[4]U.S. Department of Interior, Bureau of Land Management, Arizona State Office, Tucson, AZ, USA
[5]Arizona Institute for Resilience, University of Arizona, Tucson, AZ, USA
[6]U.S. Geological Survey, National Uncrewed Systems Office, Geosciences and Environmental Change Science Center, Denver, CO, USA
[7]U.S. Geological Survey, Geology, Geophysics, Geochemistry Science Center, Denver, CO, USA
[8]NASA Goddard Space Flight Center, Earth Sciences Division, Biospheric Sciences Laboratory, Greenbelt, MD, USA

***Corresponding author**: William K. Smith (wksmith@arizona.edu)

Acknowledgements

We acknowledge support for this research provided by the NASA Earth Surface Mineral Dust Source Investigation (EMIT) Science and Applications Team (Grant 80NSSC24K0858), the NASA Carbon Cycle Science Program (Grant 80NSSC23K0109), and the United States Geological Survey (USGS) National Land Imaging (NLI) Program. Any use of trade, firm, or product names is for descriptive purposes only and does not imply endorsement by the U.S. Government.

Contents of this file:

Supplementary Tables S1 – S8

Supplementary Figures S1 – S7

Table S1. Pairwise comparisons for ASD spectrometer ground-based estimates of chlorophyll, moisture, and brightness across biocrust functional type (BFT).

**Func Type**	**Median**	**IQR**	**LyCy**	**DkCy**	**Lichen**	**Moss**
**Chlorophyll Index**						
LtCy	0.12	0.012	--	$p < 0.001$	$p < 0.001$	$p < 0.001$
DkCy	0.15	0.037	--	--	$p < 0.001$	$p < 0.001$
Lichen	0.21	0.076	--	--	--	$p < 0.001$
Moss	0.28	0.112	--	--	--	--
**Moisture Index**						
LtCy	0.054	0.0276	--	$p < 0.001$	$p < 0.001$	$p < 0.001$
DkCy	0.077	0.0496	--	--	$p < 0.001$	$p < 0.05$
Lichen	0.149	0.0850	--	--	--	$p < 0.05$
Moss	0.137	0.0787	--	--	--	--
**Brightness Index**						
LtCy	0.16	0.015	--	$p < 0.001$	$p < 0.001$	$p < 0.001$
DkCy	0.12	0.012	--	--	$p < 0.001$	$p < 0.05$
Lichen	0.10	0.026	--	--	--	$p < 0.05$
Moss	0.11	0.017	--	--	--	--

Table S2. Pairwise comparisons for uncrewed ariel systems (UAS) estimates of chlorophyll, moisture, and brightness across biocrust functional type (BFT).

**Func Type**	**Median**	**IQR**	**Light**	**Dark**
**Chlorophyll Index**				
Light	0.116	0.0261	--	$p < 0.0001$
Dark	0.227	0.0487	--	--
**Temperature Index**				
Light	0.643	0.0398	--	$p < 0.0001$
Dark	0.558	0.0696	--	--
**Brightness Index**				
Light	0.139	0.0257	--	$p < 0.0001$
Dark	0.0781	0.0229	--	--

Table S3. Pairwise comparisons for ASD spectrometer ground-based estimates of chlorophyll, moisture, and brightness in light cyanobacteria cover across experimental treatments. Non-significant relationships denoted as “ns”.

**Treatment**	**Median**	**IQR**	**Control**	**AltP**	**Warmed**	**AltP + Warmed**
**Chlorophyll Index**						
Control	0.120	0.0187	--	ns	ns	ns
AltP	0.122	0.0131	--	--	ns	ns
Warmed	0.122	0.0157	--	--	--	ns
AltP + Warmed	0.121	0.0114	--	--	--	--
**Moisture Index**						
Control	0.0739	0.034	--	ns	ns	ns
AltP	0.0557	0.0331	--	--	ns	ns
Warmed	0.0537	0.0173	--	--	--	ns
AltP + Warmed	0.0485	0.0243	--	--	--	--
**Brightness Index**						
Control	0.153	0.0119	--	ns	ns	ns
AltP	0.153	0.0231	--	--	ns	ns
Warmed	0.155	0.0114	--	--	--	ns
AltP + Warmed	0.159	0.01	--	--	--	--

Table S4. Pairwise comparisons for ASD spectrometer ground-based estimates of chlorophyll, moisture, and brightness in dark cyanobacteria cover across experimental treatments. Non-significant relationships denoted as "ns".

**Treatment**	**Median**	**IQR**	**Control**	**AltP**	**Warmed**	**AltP + Warmed**
**Chlorophyll Index**						
Control	0.152	0.0212	--	ns	ns	ns
AltP	0.173	0.0279	--	--	ns	$p = 0.04$
Warmed	0.159	0.0242	--	--	--	ns
AltP + Warmed	0.137	0.042	--	--	--	--
**Moisture Index**						
Control	0.108	0.042	--	ns	ns	$p = 0.05$
AltP	0.0677	0.0556	--	--	ns	ns
Warmed	0.0921	0.0395	--	--	--	ns
AltP + Warmed	0.0663	0.0562	--	--	--	--
**Brightness Index**						
Control	0.115	0.0176	--	ns	ns	ns
AltP	0.116	0.00487	--	--	ns	$p = 0.02$
Warmed	0.12	0.00664	--	--	--	ns
AltP + Warmed	0.126	0.0102	--	--	--	--

Table S5. Pairwise comparisons for ASD spectrometer ground-based estimates of chlorophyll, moisture, and brightness in lichen cover across experimental treatments. Non-significant relationships denoted as “ns”.

**Treatment**	**Median**	**IQR**	**Control**	**AltP**	**Warmed**	**AltP + Warmed**
**Chlorophyll Index**						
Control	0.21	0.0589	--	ns	ns	ns
AltP	0.208	0.0654	--	--	ns	ns
Warmed	0.234	0.0814	--	--	--	ns
AltP + Warmed	0.181	0.0555	--	--	--	--
**Moisture Index**						
Control	0.175	0.0789	--	ns	ns	$p = 0.03$
AltP	0.15	0.16	--	--	ns	ns
Warmed	0.154	0.0724	--	--	--	ns
AltP + Warmed	0.108	0.0527	--	--	--	--
**Brightness Index**						
Control	0.0925	0.0203	--	ns	ns	$p = 0.03$
AltP	0.102	0.0352	--	--	ns	ns
Warmed	0.0941	0.0156	--	--	--	ns
AltP + Warmed	0.113	0.016	--	--	--	--

Table S6. Pairwise comparisons for ASD spectrometer ground-based estimates of chlorophyll, moisture, and brightness in moss cover across experimental treatments. Non-significant relationships denoted as “ns”.

Treatment	Median	IQR	Control	AltP	Warmed	AltP + Warmed
**Chlorophyll Index**						
Control	0.312	0.0236	--	p = 0.04	ns	p = 0.04
AltP	0.265	0.111	--	--	ns	ns
Warmed	0.251	0.0931	--	--	--	ns
AltP + Warmed	0.204	0.101	--	--	--	--
**Moisture Index**						
Control	0.209	0.0582	--	p = 0.02	p = 0.01	p = 0.02
AltP	0.121	0.0295	--	--	ns	ns
Warmed	0.141	0.0444	--	--	--	ns
AltP + Warmed	0.106	0.0833	--	--	--	--
**Brightness Index**						
Control	0.105	0.0154	--	ns	ns	ns
AltP	0.117	0.013	--	--	ns	ns
Warmed	0.115	0.027	--	--	--	ns
AltP + Warmed	0.115	0.012	--	--	--	--

Table S7. Pairwise comparisons for direct measurements of soil surface temperature and moisture across experimental treatments. Non-significant relationships denoted as “ns”.

Treatment	Median	IQR	Control	AltP	Warmed	AltP + Warmed
**Surface Temperature (°C)**						
Control	21.8	8.65	--	$p < 0.01$	$p < 0.01$	ns
AltP	18.3	5.01	--	--	$p < 0.0001$	$p < 0.001$
Warmed	27.2	4.38	--	--	--	ns
AltP + Warmed	27.1	4.61	--	--	--	--
**Surface Moisture (g g^{-1})**						
Control	0.0000	0.0003	--	ns	ns	ns
AltP	0.0001	0.0007	--	--	ns	ns
Warmed	0.0000	0.0001	--	--	--	ns
AltP + Warmed	0.0004	0.0008	--	--	--	--

Table S8. Pairwise comparisons for direct measurements of soil surface temperature and moisture across biocrust functional type (BFT). Non-significant relationships denoted as “ns”.

Biocrust Functional Type	Median	IQR	LtCy	DkCy	Moss
**Surface Temperature (°C)**					
LtCy	26.7	4.90	--	$p < 0.0001$	$p = 0.023$
DkCy	16.2	5.30	--	--	$p < 0.001$
Moss	21.8	6.02	--	--	--
**Surface Moisture (g g^{-1})**					
LtCy	0.0001	0.0008	--	ns	ns
DkCy	0.0002	0.0007	--	--	ns
Moss	0.0000	0.0002	--	--	--

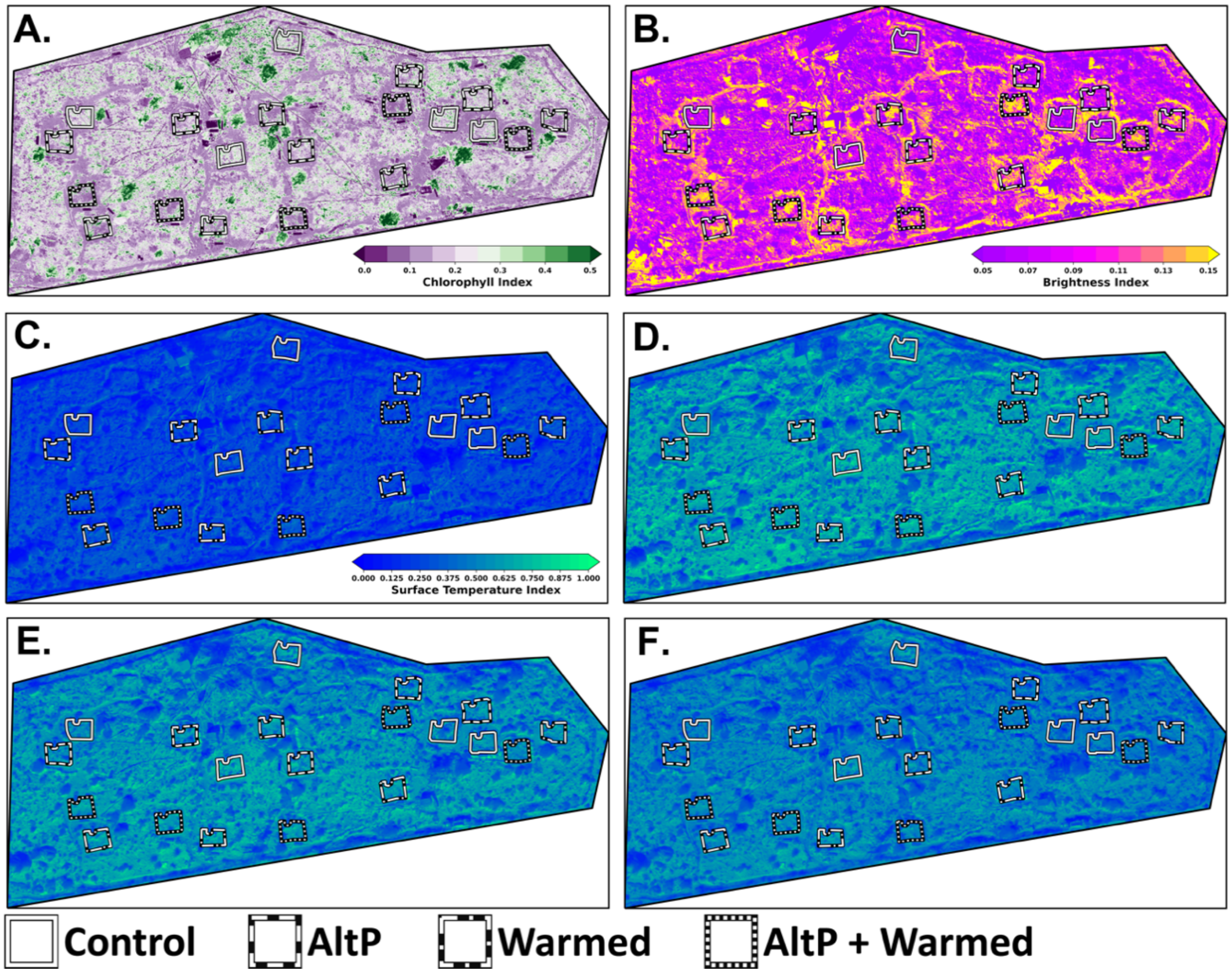

Figure S1. Uncrewed aerial system (UAS) measured chlorophyll, brightness, and surface temperature index. Map of experimental site shown in each panel and outlines of plots can be seen in each maps. (A) shows chlorophyll index, (B-F) show surface brightness index at 10:33, 12:47, 13:58, and 15:58 respectively.

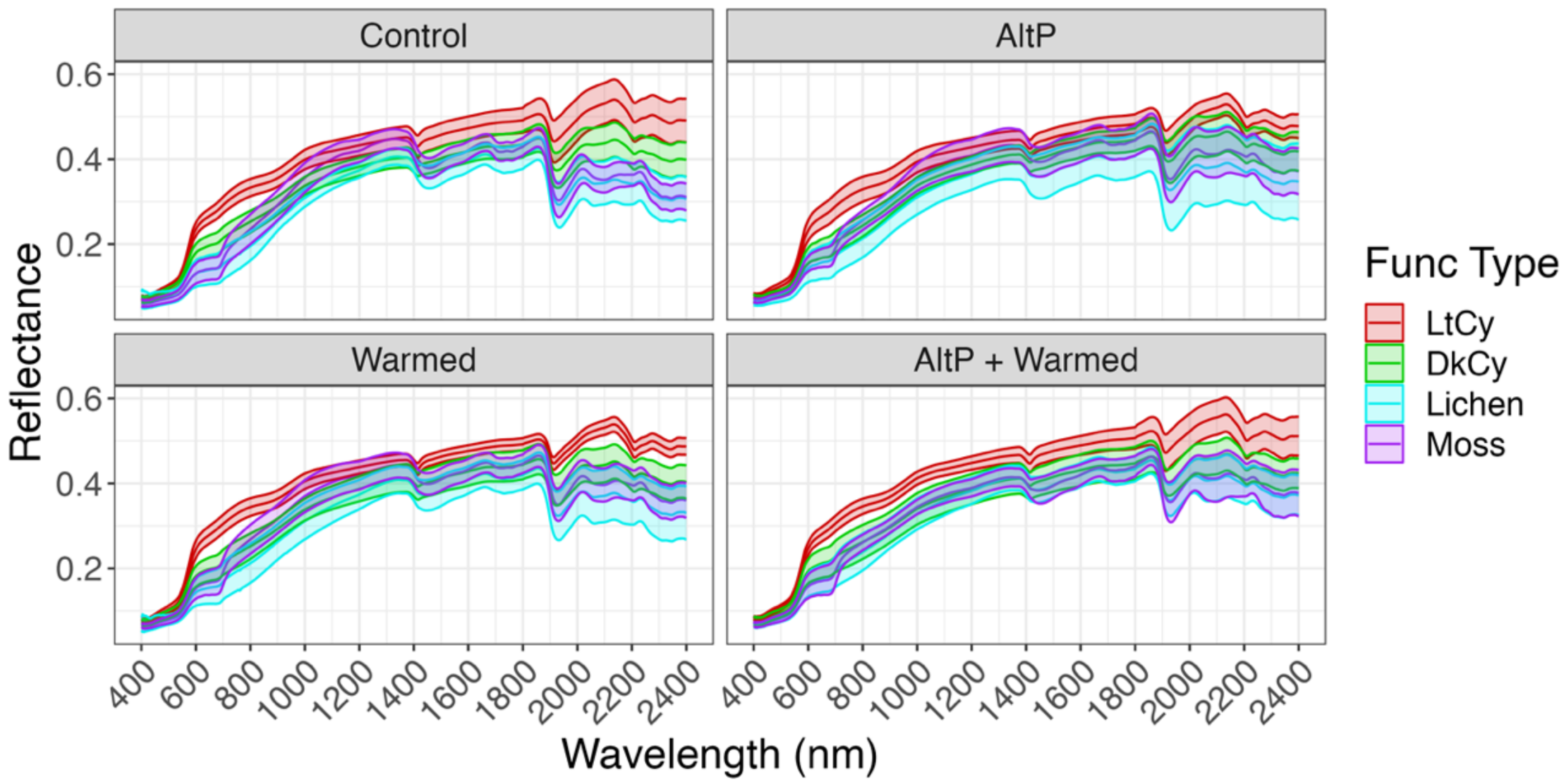

Figure S2. Field-based spectral signatures of isolated biocrust functional types (BFTs) measured across all experimental plots and aggregated by treatment. Top rows show treatments without experimental warming (control and altered precipitation) and bottom rows show treatments with warming (warmed and altered precipitation + warmed).

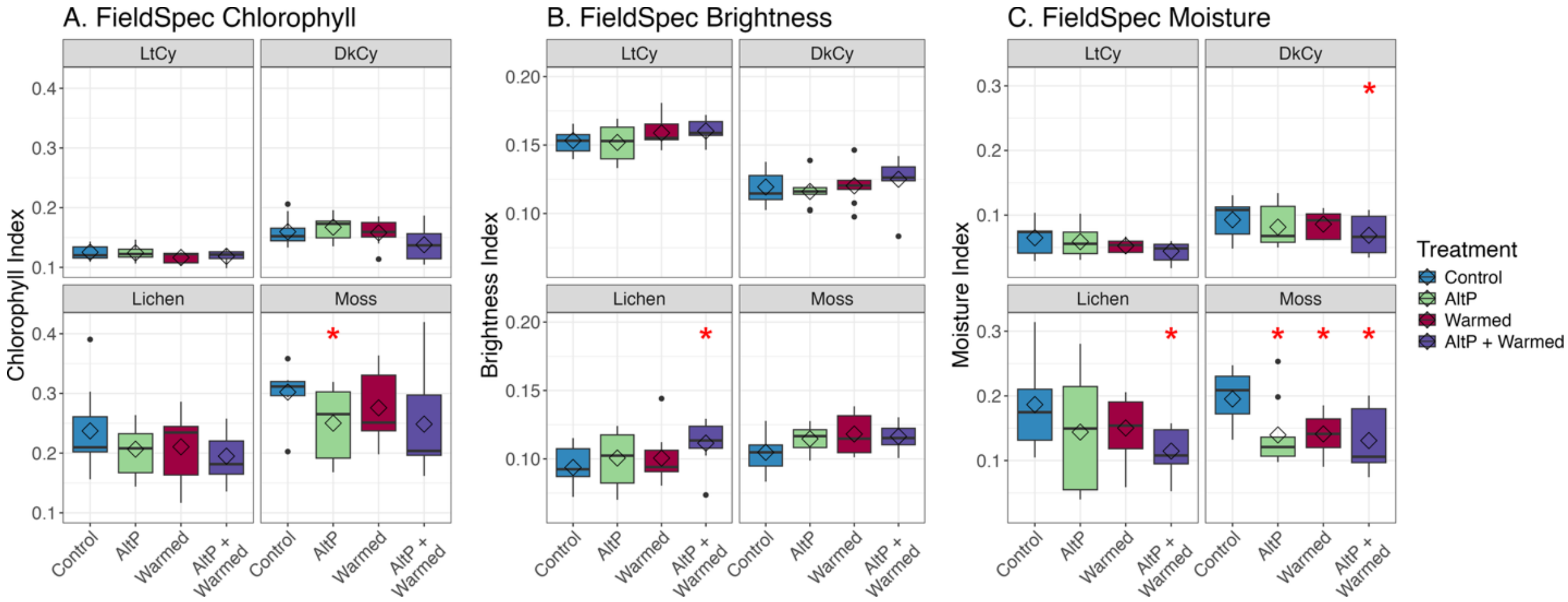

Figure S3. Field-measured chlorophyll, brightness, and water indices as a function of treatment. Experimental treatments are shown in each panel and included control (Control) altered precipitation (AltP), warming (Warmed), and altered precipitation and warming combined (AltP + Warmed) treatments. Statistical significance from the Control plots is shown as * = $p < 0.05$; ** = $p < 0.01$; and *** = $p < 0.001$.

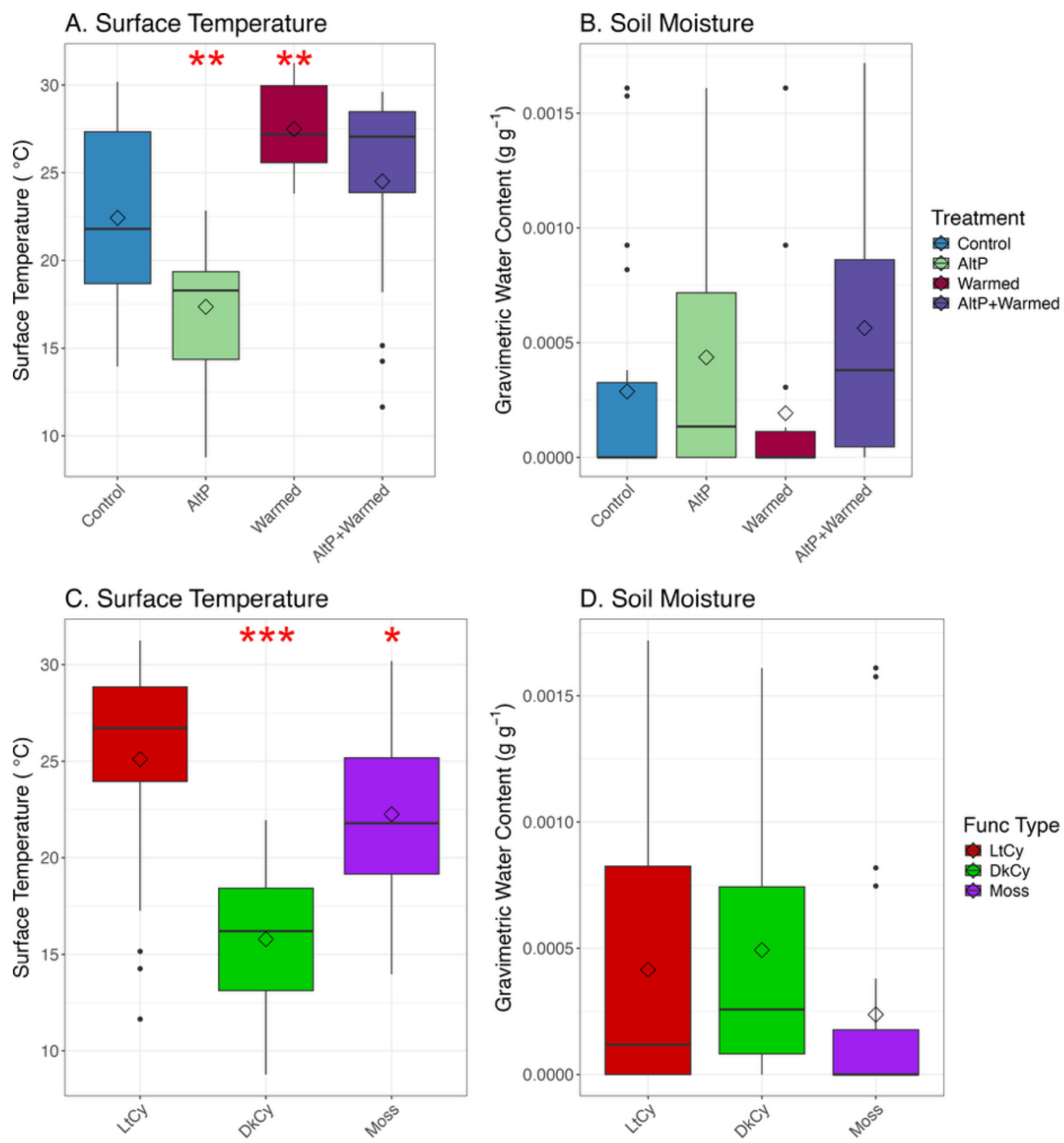

Figure S4. Direct measurements of surface temperature (A, C) and soil moisture (B, D) by treatment (A, B) and biocrust functional type (BFT) (C, D). Statistical significance is shown as * = $p < 0.05$; ** = $p < 0.01$; and *** = $p < 0.001$.

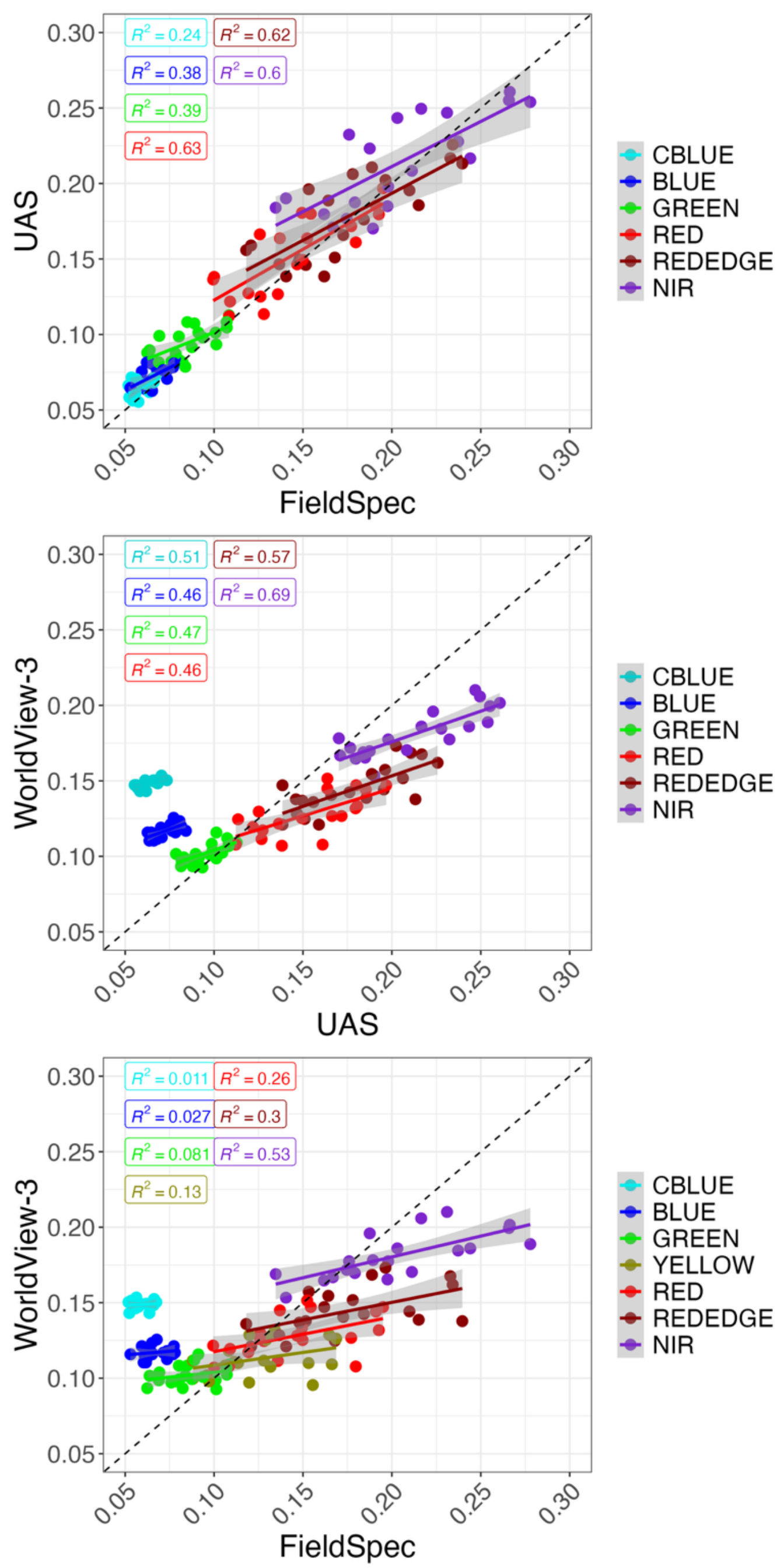

Figure S5. The relationship between multi-scale remotely sensed surface reflectance estimates across all experimental plots. ASD spectrometer versus uncrewed aerial system (UAS) estimates of surface reflectance across all plots and shared spectral bands (A). UAS versus satellite WorldView-3 estimates of surface reflectance across all plots and shared spectral bands (B). ASD spectrometer versus satellite WorldView-3 estimates of surface reflectance across all plots and shared spectral bands (C). R^2 represents the proportion of the variance for a given spectral band that's explained by the independent variable.

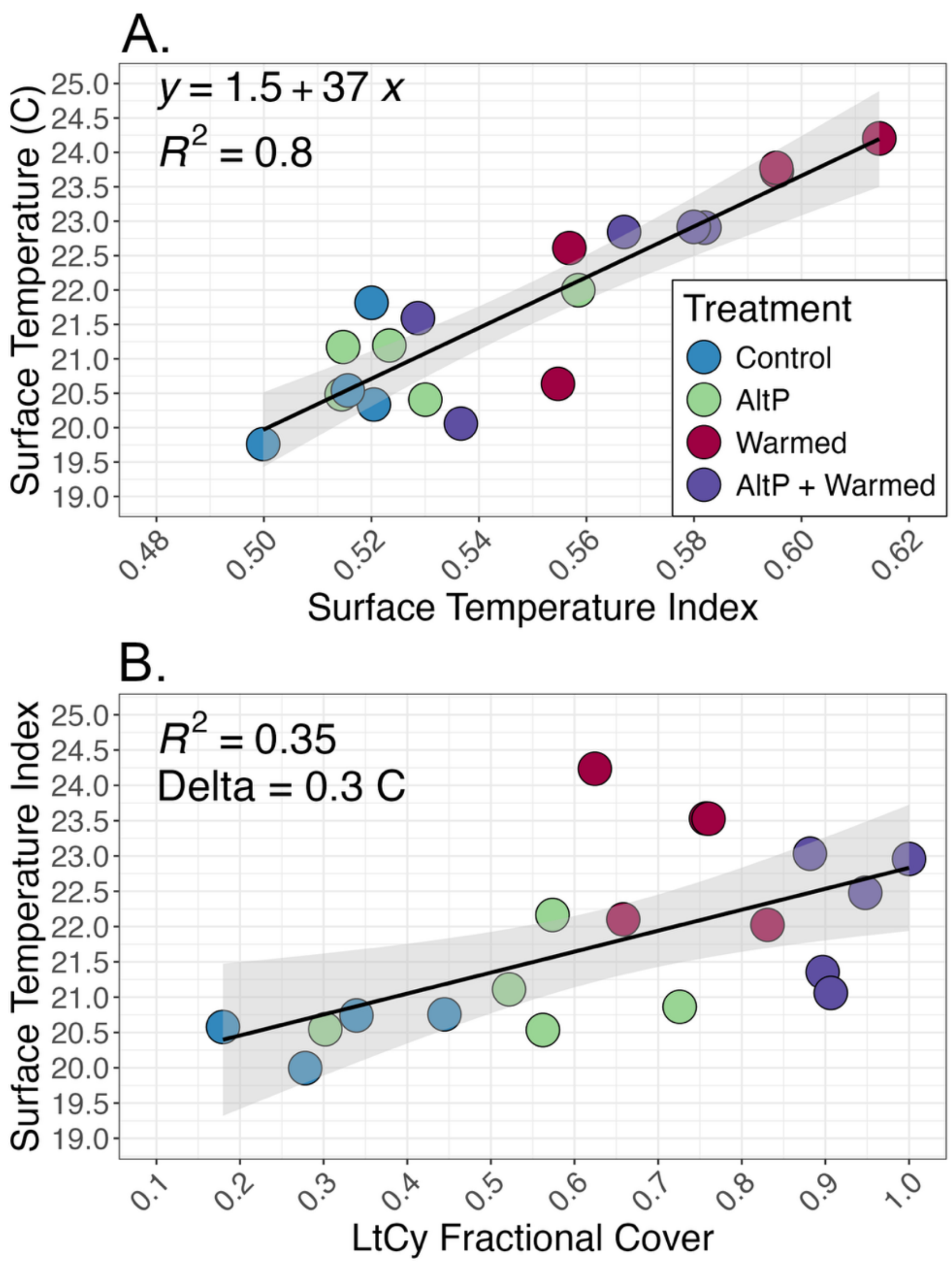

Figure S6. The relationship between remotely sensed relative and absolute surface temperature across all experimental plots (A). The relationship between absolute surface temperature and light cyanobacteria (LtCy) fractional cover across all experimental plots (B). R^2 represents the proportion of the variance for a given climate regulation index that's explained by vascular plant fractional cover. Delta represents the percentage change in absolute surface temperature per 0.1 change in light cyanobacteria fractional cover.

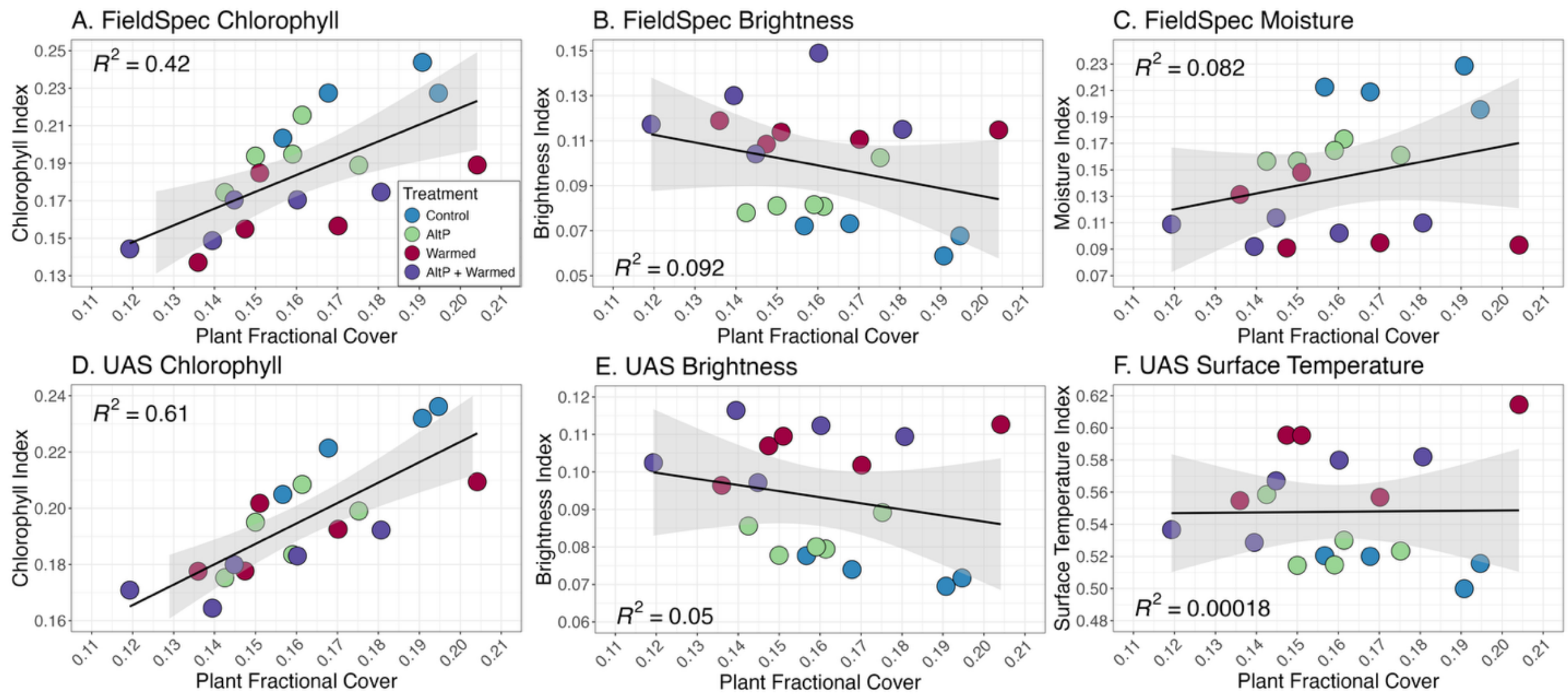

Figure S7. The relationship between remotely sensed functional indices and vascular plant (Plant) fractional cover across all experimental plots. The top row (A – C) shows ASD FieldSpec estimates of chlorophyll (A), brightness (B), and moisture (C) indices and their relationship with vascular fractional cover. The bottom row (D – F) shows UAS estimates of chlorophyll (A), brightness (B), and temperature (C) indices and their relationship with vascular plant fractional cover. Each circle in the plot represents a single plot and is colored by treatment: control (Control) altered precipitation (AltP), warming (Warmed), and altered precipitation and warming combined (AltP + Warmed). R2 represents the proportion of the variance for a given climate regulation index that's explained by vascular plant fractional cover.
