# Supplementary material for: Experimental evidence of a biological soil crust degradation climate warming amplification feedback
Source: Commun Earth Environ. 2026 Sep 12;7(1):775. doi: 10.1038/s43247-026-03874-5 (PMC13593566; doi:10.1038/s43247-026-03874-5)
Supplement: Supplementary file 2 [file 43247_2026_3874_MOESM2_ESM.pdf]

Peer Review File

Experimental evidence of a biological soil crust degradation climate warming amplification feedback

Corresponding Author: Professor William Smith

This file contains all reviewer reports in order by version, followed by all author rebuttals in order by version.

Version 0:

Reviewer comments:

Reviewer #1

(Remarks to the Author)
The study integrates in-situ climate manipulation experiments with multi-scale remote sensing observations, leveraging nearly two decades of continuous monitoring data and multi-source spatial data. It systematically investigates the core scientific questions concerning "the mechanisms by which climate change affects biocrust community structure, the consequent changes in their climate regulation functions, and the resulting feedback effects on global warming." The findings reveal that climate warming drives biocrust degradation from late-successional types such as mosses towards early-successional types like lightly-pigmented cyanobacteria. This significantly reduces their photosynthetic carbon sequestration capacity, surface moisture retention, and cooling effects, thereby forming a positive feedback loop of "biocrust degradation-climate warming amplification." This research provides crucial theoretical foundations and methodological support for refining biocrust feedback mechanisms in Earth system models, formulating dryland ecological conservation strategies, and establishing a global biocrust dynamic monitoring system.
However, several issues in the manuscript may require clarification or revision before publication. Specific comments are as follows:
Lines 190-191: Why were there no surface temperature measurements specifically targeting lichen biocrusts at the experimental site? Was this due to experimental constraints, or is it because lichen biocrusts, as an intermediate successional stage, might be considered negligible?
Line 244: It is recommended to add a legend for circle sizes in Figure 3 to more intuitively illustrate the coverage levels of vascular plants.
Lines 253-254: What are the specific criteria for classifying the coverage levels of vascular plants (very low, low, medium, high)?
Lines 279-280: Given the acknowledged limitations in the surface moisture measurements in this study, it is advisable to seek and cite supporting evidence from other relevant literature to substantiate this statement.
Lines 466-468: What was the rationale behind selecting the 2°C and 4°C temperature increases for the warming experiment? Is maintaining the same level (4°C) from 2008 to 2022 without adjustment justifiable?
Lines 522, 554, 601: For the three data acquisition and processing approaches (field, UAS, satellite), were specific considerations given to the issue of vascular plant occlusion over biocrusts in biocrust remote sensing?
Lines 697, 724, 802, 806: Please correct "CO2" to "CO_2".
Lines 776-777: Please add the DOI: https://doi.org/10.5751/ES-02412-130228.
Lines 839-840: Please add the DOI: https://doi.org/10.1111/nph.13949.

Reviewer #2

(Remarks to the Author)
This manuscript examines how long-term climate change alters biological soil crust (biocrust) communities and how these shifts may feed back to local climate. Using a two-decade dryland climate manipulation experiment combined with multi-scale remote sensing, the authors assess the effects of experimental warming (+4 °C) and altered monsoonal rainfall on biocrust composition and associated ecosystem processes. They report a decline in late-successional moss-dominated crusts and an increase in early-successional lightly pigmented cyanobacteria, changes that are linked to reduced photosynthetic potential, lower surface moisture, and higher surface temperatures.
The study addresses an important and timely question because biocrusts cover a substantial fraction of global drylands and

play key roles in regulating carbon uptake, soil moisture, and surface energy balance. Understanding how climate-driven changes in biocrust communities influence land–atmosphere feedbacks is therefore critical, particularly as such processes are currently underrepresented in climate and ecosystem models.

The manuscript presents a robust experimental design, a valuable dataset, and statistically significant results, clearly documenting climate-driven shifts in biocrust community composition and associated changes in surface properties. To further strengthen the manuscript, it would be helpful to more explicitly highlight how these findings advance beyond previous studies, for example by clarifying the novel aspects of the long-term experiment, the integration of multi-scale remote sensing, or the quantification of feedbacks to surface temperature. Additionally, while the use of remote sensing to detect community shifts is compelling, the discussion could be expanded to illustrate the broader implications or potential applications of this approach, such as large-scale monitoring of dryland ecosystems or integration into predictive models. These clarifications would help readers better appreciate both the scientific contribution and practical relevance of the study.

The connection between the observed compositional changes and the ecosystem's functional shifts is not fully articulated. Although the authors report changes in photosynthetic potential, surface moisture, and temperature, the ecological and biogeochemical consequences of these shifts are not discussed in detail. Expanding on how the documented community transitions translate into broader functional changes in dryland systems would clarify the study's significance and potential applications.

The manuscript integrates measurements from several sensing platforms (handheld spectroradiometer, UAS multispectral/thermal imagery, and WorldView-3 satellite imagery), which is a strength of the study. However, it is not fully clear how measurements collected at these different spatial scales are made comparable. In particular, the plot-scale ground spectra (ASD FieldSpec measurements) represent very small sampling footprints and high spectral resolution (1 nm), whereas the satellite observations (WorldView-3) have a spatial resolution of ~1.24 m and only eight broad spectral bands. Similarly, the UAS imagery has centimeter-level spatial resolution and different spectral characteristics. While the manuscript states that pixels were aggregated to the plot scale for the satellite data, it remains unclear how the authors ensured comparability between these datasets in terms of spatial footprint, spectral band alignment, and radiometric consistency. The authors should clarify the scaling strategy used to link ground measurements, UAS observations, and satellite imagery. For example, it would be helpful to explain whether ground spectra were convolved to the spectral response functions of the airborne/satellite sensors, how spatial heterogeneity within plots was handled when aggregating pixels, and how uncertainties introduced by the scale mismatch were assessed. Without a clearer description of these steps, it is difficult to evaluate the robustness of the multi-scale remote sensing comparison.

As satellite observations can reproduce or approximate plot-scale patterns observed in the field (which still needs to be validated for comparison, see my previous comment), the manuscript should further explore the temporal potential of satellite data. Specifically, once the relationship between plot measurements and satellite reflectance is established, historical satellite archives could be used to examine how these plots may have changed over time. Such an analysis could offer valuable context for the experimental results by placing the observed community shifts within a longer-term perspective and evaluating whether the detected biocrust changes align with broader trends observable at the site level. Even a preliminary analysis of such time series could significantly strengthen the manuscript by demonstrating the potential for long-term monitoring of biocrust dynamics from space and by emphasising the practical relevance of satellite remote sensing beyond the timeframe of field experiments.

Minor comments:

Figure 1a: The location of the study site is not immediately clear. Consider providing an improved map with clearer boundaries, labels, and scale to help the reader orient themselves.
Lines 166–167: The text states that only one cyanobacterial BFTs showed no differences between treatments for all indices, but Fig. S3 is not fully reflected in this description (both cyanobacterial BFTs show no differences). Consider revising the text to more accurately summarize what the figure shows, so that readers can clearly see the correspondence between the results and the figure.

Version 1:

Reviewer comments:

Reviewer #1

(Remarks to the Author)
The study integrated in-situ climate manipulation experiments with multi-scale remote sensing observations, leveraging nearly two decades of continuous monitoring data and multi-source spatial data. It systematically investigated the core scientific questions concerning "the mechanisms by which climate change affects biocrust community structure, the consequent changes in their climate regulation functions, and the resulting feedback effects on global warming."
The main results showed that two decades of in situ climate warming and changes in monsoonal rainfall drove on average a

~27% decrease in fractional coverage of late-successional biocrust mosses and a ~36% increase in fractional cover of early-successional lightly-pigmented cyanobacteria.
The findings provided evidences that the biocrust degradation climate warming amplification feedback, whereby climate warming is driving biocrust community composition shifts and functional degradation and a net reduction in climate mitigation potential, creating a feedback loop that further amplifies warming.This apparent amplification feedback is currently missing from process-based models, and thus it's likely that current climate projections from these models underestimate the rate of climate warming across global drylands.
So, this research provides crucial theoretical foundations and methodological support for refining biocrust feedback mechanisms in Earth system models, formulating dryland ecological conservation strategies, and establishing a global biocrust dynamic monitoring system.

I have checked all the comments from the two reviews to the manscript, and found that the authors have carefully considered all the comments. The manscript is very strong now.

However, there still have a question that the biological soil crust communities at different succession stages respond differently to climate change. Therefore, what are the response mechanisms of the biological soil crusts at different succession stages to climate change? What are the similarities and differences? Especially in Figure 5, I think this concept diagram is a bit simplistic and lacks the description of the process.

Reviewer #2

(Remarks to the Author)
I would like to thank the authors for their thorough revision. The MS has greatly improved.
I don't have further specific comments.

Minor comment:
Fig. 5: The text in red in the picture (left picture) is hard to read. Please consider using another colour.

Open Access This Peer Review File is licensed under a Creative Commons Attribution 4.0 International License, which permits use, sharing, adaptation, distribution and reproduction in any medium or format, as long as you give appropriate credit to the original author(s) and the source, provide a link to the Creative Commons license, and indicate if changes were made.
In cases where reviewers are anonymous, credit should be given to 'Anonymous Referee' and the source.
The images or other third party material in this Peer Review File are included in the article's Creative Commons license, unless indicated otherwise in a credit line to the material. If material is not included in the article's Creative Commons license and your intended use is not permitted by statutory regulation or exceeds the permitted use, you will need to obtain permission directly from the copyright holder.

To view a copy of this license, visit https://creativecommons.org/licenses/by/4.0/

Experimental evidence of a biological soil crust degradation climate warming amplification feedback

Response to Reviewers

Editor (Remarks to the Author):

Your manuscript titled "Experimental evidence of a biological soil crust degradation climate warming amplification feedback" has now been seen by 2 reviewers, and we include their comments at the end of this message. They find your work of interest, but some important points are raised. We are interested in the possibility of publishing your study in Communications Earth & Environment, but would like to consider your responses to these concerns and assess a revised manuscript before we make a final decision on publication.

[Author response] We greatly appreciate the constructive comments from the editor and reviewers, which we closely followed to thoroughly revise our manuscript. Please see below our point-by-point responses in blue text following each comment. Note, the line numbers in our responses are for the clean (non-tracked-changes) version of the revised manuscript.

[Editor, Comment 1] Clearly present sufficient novelty into the mechanism of climate change-driven shifts in biological soil crust community affect their climate mitigation potential, and create feedback loops that may amplify future warming.

[Author response] We have now added additional results that further demonstrate the novelty and robustness of our findings. We have moved our results based on WorldView-3 data to the main text, which demonstrate that we are able to capture treatment effect using very high resolution satellite data (new Fig. 4). We additionally added an evaluation of our scaling framework that clearly demonstrates that plot-level Fieldspec, UAS, and WorldView-3 remote sensing observations are correlated (see new Fig. S5 in response to Reviewer #2) and capture treatment effects – climate warming and altered precipitation are driving biocrust community composition shifts and functional degradation and a net reduction in climate mitigation potential. Finally, we have also added a more rigorous analysis that confirms the change in plot-level climate mitigation potential is being dominantly driven by biocrusts and not vascular plants (Fig. S7).

[Editor, Comment 2] Clearly present the broader implication of the study.

[Author response] We now add additional text to the abstract (Lines 34 – 42) and a new final paragraph to the discussion that underscores the novelty of our findings and the uniqueness and potential adaptability of the approach (Lines 469 – 486). We include the revised text with changes italicized and underlined below for reviewer convenience.

Lines 34 – 42:
"Our findings are evidence of a biocrust degradation climate warming amplification feedback, whereby climate warming is driving biocrust community composition shifts and functional degradation and a net reduction in climate mitigation potential, *creating a feedback loop that*

further amplifies warming. This apparent amplification feedback is currently missing from process-based models, and thus it's likely that current climate projections from these models underestimate the rate of climate warming across global drylands. Our work also underscores the need for expanded monitoring, restoration, and protection of late-successional, darkly-pigmented biocrusts across global drylands for their significant role in ecosystem function and climate mitigation."

Lines 469 – 486:

"***Enhancement of global change experiments through the integration of remote sensing***

We demonstrate an approach in which we integrate multiscale remote sensing measurements (field, UAS, and satellite) within a long-term global change experiment to reveal a novel biocrust degradation – climate warming amplification feedback that is likely already occurring across vast global dryland ecosystems. Further, our approach enabled a nuanced enhancement to previous efforts, which by focusing only on albedo, came to an opposite conclusion – expansion of early-successional light cyanobacteria and the loss of late-successional mosses could result in surface cooling due to albedo increases (Rutherford et al., 2017). Our more comprehensive view was achievable by the integration of multiscale remote sensing measurements over the optical and thermal regions of the electromagnetic spectrum, which revealed substantial increases in surface temperature in climate change treatment plots, despite major increases in light cyanobacteria fractional coverage and corresponding increases in surface brightness (Figure 3, 5). These findings underscore the need to monitor, restore, and protect late-successional biocrusts across global drylands for their significant role in ecosystem function and climate mitigation. The approach developed here could be easily adapted for application in a wide range of existing long-term global change experiments to complement traditional methods and extend the inferences gained from these critically important yet time-, labor-intensive, and expensive undertakings (Shiklomanov et al., 2019)."

Reviewer #1 (Remarks to the Author):

The study integrates in-situ climate manipulation experiments with multi-scale remote sensing observations, leveraging nearly two decades of continuous monitoring data and multi-source spatial data. It systematically investigates the core scientific questions concerning "the mechanisms by which climate change affects biocrust community structure, the consequent changes in their climate regulation functions, and the resulting feedback effects on global warming." The findings reveal that climate warming drives biocrust degradation from late-successional types such as mosses towards early-successional types like lightly-pigmented cyanobacteria. This significantly reduces their photosynthetic carbon sequestration capacity, surface moisture retention, and cooling effects, thereby forming a positive feedback loop of "biocrust degradation-climate warming amplification." This research provides crucial theoretical foundations and methodological support for refining biocrust feedback mechanisms in Earth system models, formulating dryland ecological conservation strategies, and establishing a global biocrust dynamic monitoring system. However, several issues in the manuscript may require clarification or revision before publication.

[Author response] We sincerely thank the reviewer for their positive and constructive feedback,

which has helped us to improve the manuscript. Please see below our point-by-point responses in blue text following each reviewer comment. Note, the line numbers in our responses are for the clean (non-tracked-changes) version of the revised manuscript.

[Reviewer #1, Comment 1] Lines 190-191: Why were there no surface temperature measurements specifically targeting lichen biocrusts at the experimental site? Was this due to experimental constraints, or is it because lichen biocrusts, as an intermediate successional stage, might be considered negligible?

[Author response] The reviewer highlights here an important experimental constraint. Unfortunately, we were only able to collect thermal images of surface temperature at the uncrewed aerial system (UAS)-scale. At the UAS-scale, it was not feasible to separate the different late successional biocrusts functional types (i.e., dark cyanobacteria, lichens, and mosses) because the lichens are primarily darkly pigmented (*Collema tenax* and *C. coccophorum*) and are similar in color to mosses and darkly pigmented cyanobacteria. Thus, instead, we grouped the biocrust communities into early (i.e., lightly pigmented cyanobacteria) and late (i.e., darkly pigmented cyanobacteria, lichens, and mosses) successional functional types. We have now added text to clarify this limitation on Lines 562 – 567. We have included the revised text with changes italicized and underlined below for reviewer convenience.

Lines 562 – 567:
"As part of this campaign, we collected ground-based hyperspectral data at the BFT and plot level using a handheld spectroradiometer. We also collected uncrewed aerial system (UAS) natural color red-green-blue (RGB), 10-band multispectral, and thermal infrared data at the BFT and plot scale. <u>*We note that due to the relatively coarse scale of the aerial UAS collection, BFT were grouped more broadly into early-successional (light cyanobacteria) and late-successional (dark cyanobacteria, lichens, and mosses) functional types*</u>."

[Reviewer #1, Comment 2] Line 244: It is recommended to add a legend for circle sizes in Figure 3 to more intuitively illustrate the coverage levels of vascular plants.

[Author response] This is a great point. We explored adding vascular plants fractional coverage to the figure legend, but we felt it made the figure unnecessarily complicated. Alternatively, we decided to remove vascular plant fractional coverage from the revised version of Figure 3 and instead add a new supplementary figure focused on vascular plant fractional cover (revised Figure S5). With this revision, we much more clearly highlight biocrusts as the dominant factor driving climate mitigation at the plot level. This also allowed some additional insights into the secondary role that vegetation is playing in climate mitigation. We have now revised the results and discussion to highlight these new insights (Lines 233 – 239 and Lines 355 – 357). We have included the revised text and figures with changes italicized and underlined below for reviewer convenience.

Lines 233 – 239:
"<u>*Repeating this analysis with a focus on vascular plant fractional cover, we found a relatively minor role of vascular plants in driving changes in climate mitigation potential across treatments*</u>

(Fig. S7). Vascular plant fractional cover was found to have a substantial impact on plot chlorophyll and explained 42% and 61% of the variance in the chlorophyll index for the ASD FieldSpec- and UAS-based estimates, respectively (Fig. S7A,D). By contrast, vascular plant fractional cover was not a significant factor in explaining plot-level differences in surface brightness (Fig. S7B,E), surface moisture (Fig. S7C), or surface temperature (Fig. S7F)."

Lines 355 – 357:

"*Importantly, we further show that while vascular plant cover varied significantly across plots, it explained relatively less of the variance in functional traits compared to the biocrusts* (Fig. S7)."

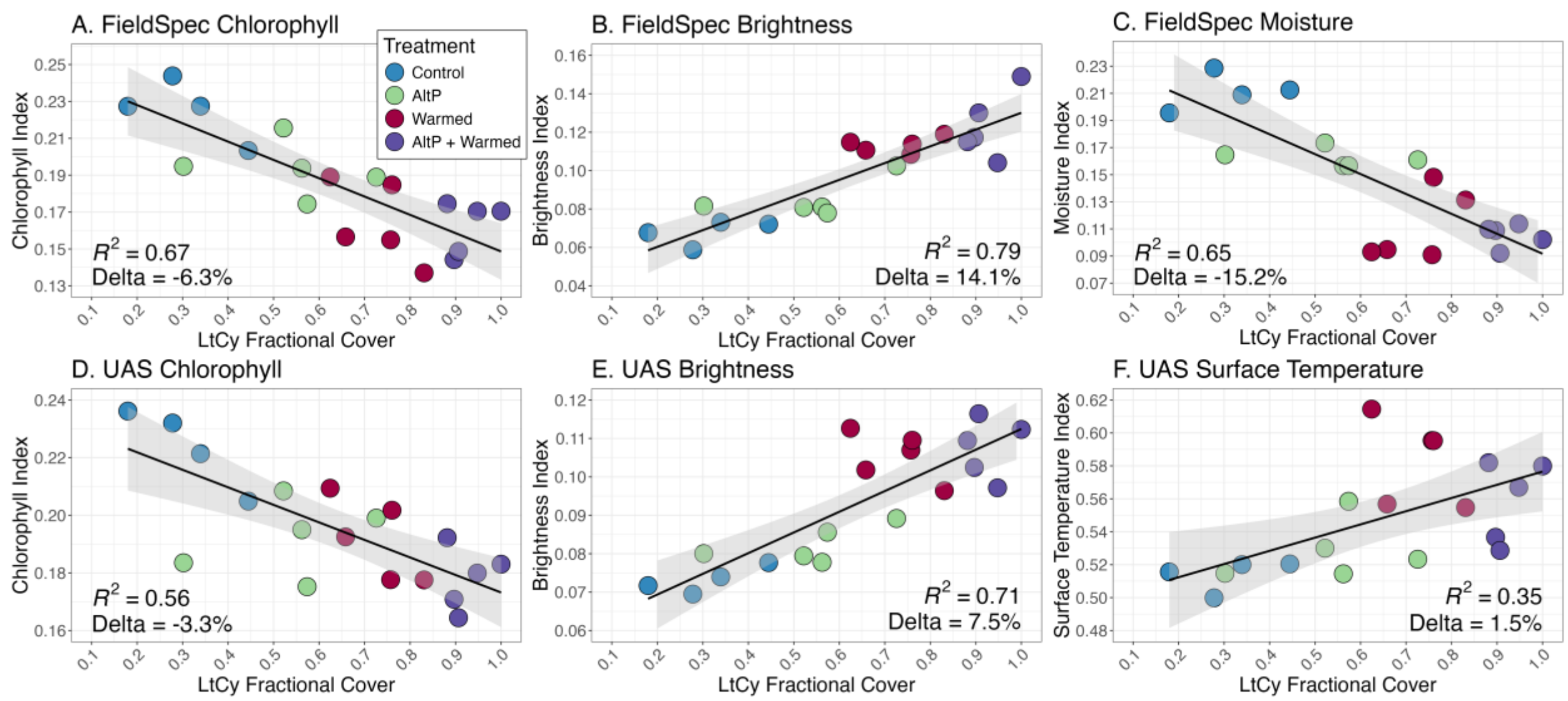

Figure 3. The relationship between remotely sensed functional indices and lightly-pigmented cyanobacteria (LtCy) fractional cover across all experimental plots. The top row (A – C) shows ASD FieldSpec estimates of chlorophyll (A), brightness (B), and moisture (C) indices and their relationship with light cyanobacteria fractional cover. The bottom row (D – F) shows UAS estimates of chlorophyll (A), brightness (B), and temperature (C) indices and their relationship with light cyanobacteria fractional cover. Light cyanobacteria fractional cover was estimated as the ratio of light cyanobacteria cover to the sum of total BFT cover (e.g., the sum of light cyanobacteria, dark cyanobacteria, lichen, and moss cover). Each circle in the plot represents a single plot and is colored by treatment: control (Control) altered precipitation (AltP), warming (Warmed), and altered precipitation and warming combined (AltP + Warmed). R^2 represents the proportion of the variance for a given climate regulation index that's explained by lightly-pigmented cyanobacteria fractional cover. Delta represents the percentage change for a given climate regulation index per 0.1 change in light cyanobacteria fractional cover. All relationships (A – F) are significant at a $p < 0.0001$ level.

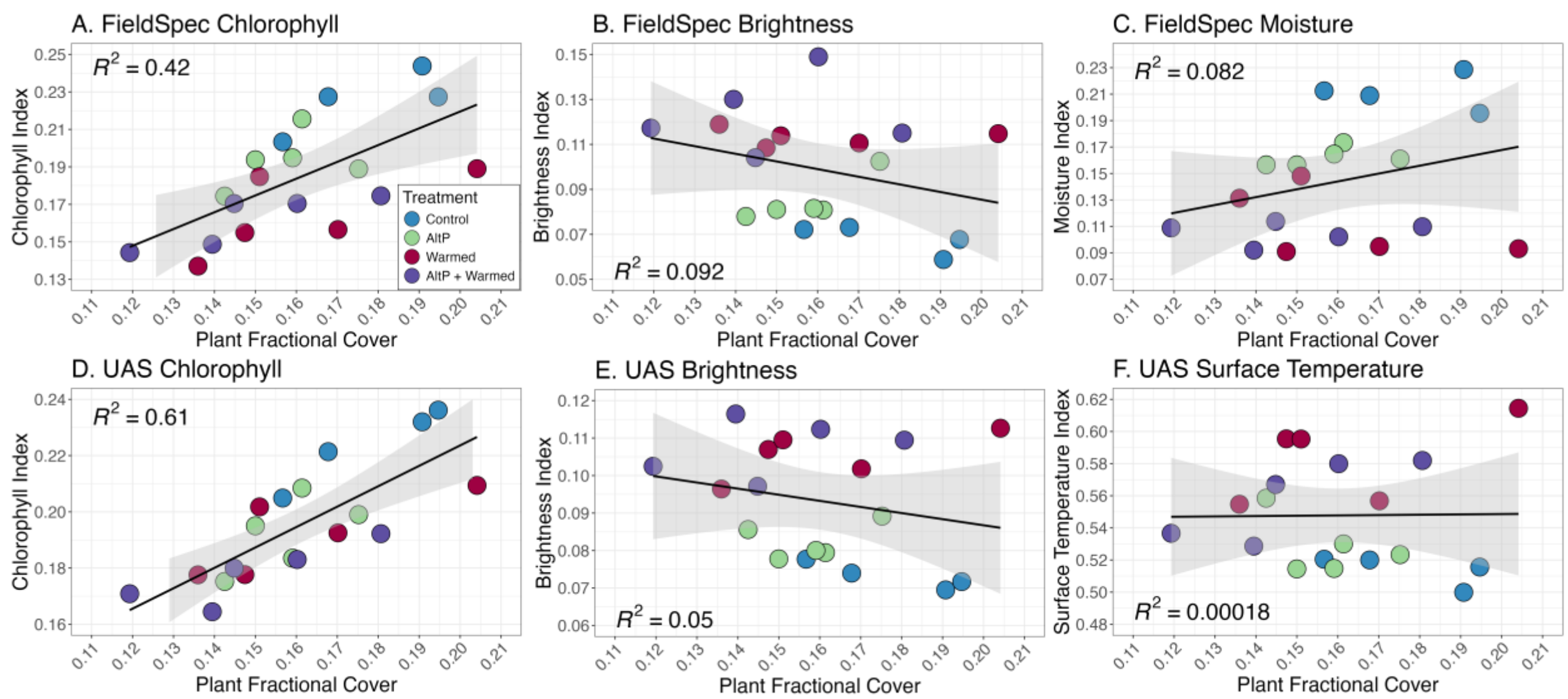

Figure S7. The relationship between remotely sensed functional indices and vascular plant (Plant) fractional cover across all experimental plots. The top row (A – C) shows ASD FieldSpec estimates of chlorophyll (A), brightness (B), and moisture (C) indices and their relationship with vascular fractional cover. The bottom row (D – F) shows UAS estimates of chlorophyll (A), brightness (B), and temperature (C) indices and their relationship with vascular plant fractional cover. Each circle in the plot represents a single plot and is colored by treatment: control (Control) altered precipitation (AltP), warming (Warmed), and altered precipitation and warming combined (AltP + Warmed). R^2 represents the proportion of the variance for a given climate regulation index that's explained by vascular plant fractional cover.

[Reviewer #1, Comment 3] Lines 253-254: What are the specific criteria for classifying the coverage levels of vascular plants (very low, low, medium, high)?

[Author response] We have now replaced this categorical classification with a much more quantitative approach (Fig. S7). With this revision, we much more clearly highlight biocrusts as the dominant factor regulating climate mitigation at the plot level. Please see our response to Reviewer #1, Comment 2 for more detail on this change.

[Reviewer #1, Comment 4] Lines 279-280: Given the acknowledged limitations in the surface moisture measurements in this study, it is advisable to seek and cite supporting evidence from other relevant literature to substantiate this statement.

[Author response] Great point. We have now added in relevant references on Lines 299 – 301. We go on, in the next paragraph, to discuss in detail the findings of these supporting manuscripts (Lines 302 – 321). We include the revised text with changes italicized and underlined below for reviewer convenience.

Lines 299 – 301:
"In particular, the cooler soil temperatures were associated with wetter soils, with connections to the effects different biocrust communities have on dryland hydrology *(Stoy et al., 2012; Chen et*

al., 2019; Xiao and Bowker, 2020)."

Lines 302 – 321:
"Our findings are in support of previous research that has found mosses have the highest rates of photosynthesis (Qiu et al., 2024; Román et al., 2019) and surface moisture (Xiao and Bowker, 282 2020) and the lowest surface temperatures (Xiao and Bowker, 2020) relative to other BFTs. Chen et al., (2019) provided evidence that the higher surface area to volume ratio of mosses resulted in greater rates of surface evaporation, which could explain the relatively high surface moisture and reduced surface temperature indices that we found in our study."

[Reviewer #1, Comment 5] Lines 466-468: What was the rationale behind selecting the 2℃ and 4℃ temperature increases for the warming experiment? Is maintaining the same level (4℃) from 2008 to 2022 without adjustment justifiable?

[Author response] We started with 2 °C warming following the internationally recognized threshold associated with the IPCC of 1.5 – 2 °C of warming as a critical range for avoiding he most severe climate impacts (i.e. RCP2.6 scenario), which is currently viewed as the most optimistic outlook for future warming. In 2008, we increased warming to 4 °C above ambient to represent a high-end warming scenario consistent with earlier high-emissions projections (i.e. RCP8.5 scenario). The site is in Southeastern, Utah, where warming is already at the higher end of current warming estimates as temperatures have already risen more than 2.5 °C since the beginning of the 20th century and are projected to reaching over 5°C by 2100 according to the NOAA Utah State Climate Summary 2022 (Kunkel et al., 2022). For these reasons, the 4°C warming treatment remains useful for evaluating dryland ecosystem responses and ecological thresholds under extreme warming scenarios and is a realistic upper bound for the potential future conditions at this site and ecosystem that is consistent with both global and regional estimates of climate projections. We have now updated the methods section to include these details. We also now clarify that background variations in temperature continue to provide realistic year-to-year variation in temperature (Lines 507 – 513). We include the revised text with changes italicized and underlined below for reviewer convenience.

Lines 507 – 513:
"The warming treatment began in October 2005 and raised soil surface temperatures by 2 °C above ambient. In June 2008, the warming treatment was adjusted to 4 °C above ambient to better align with updated climate projections for the region *(Lee et al., 2023; Schwinning, et al. 2008; Smith et al., 2005). Southeast Utah, the location of the research site, has already experienced relatively high-end warming, with updated projections of 5°C warming by 2100 (Kunkel et al., 2022). In addition to the warming treatment, background variations in temperature continue to provide realistic year-to-year variation in temperature."*

[Reviewer #1, Comment 6] Lines 522, 554, 601: For the three data acquisition and processing approaches (field, UAS, satellite), were specific considerations given to the issue of vascular plant occlusion over biocrusts in biocrust remote sensing?

[Author response] Vascular plant occlusion over biocrusts is an important source of noise in our analysis. The point frame fractional cover estimates considered multi-layer vegetation and thus

includes instances of biocrust cover under plant canopies. By contrast, the remote sensing techniques that we used mostly measured the vascular plants only in these cases. We now acknowledge this source of noise in the revised manuscript (Lines 535 - 539). Ultimately, based on the strong relationships between biocrust fractional cover and changes in climate mitigation potential (Fig. 3), we argue that this source of noise did not have a major impact on our findings. That said, our findings might in fact be conservative since removal of this noise would likely further strengthen biocrust – climate regulation relationships. We are currently collecting additional data that account for this source of noise that will be the basis for follow up work on this topic. We include the revised text with changes italicized and underlined below for reviewer convenience.

Lines 535 - 539:
"*We note that biocrusts found under vascular plant canopies were counted in the point intercept-based estimates of fractional cover. This introduced an unavoidable source of uncertainty in our comparison of plot-level biocrust fractional cover and remote sensing-based climate mitigation potential estimates.* Further details on the method used for estimating plot-level fractional cover can be found in Reed et al., (2012)."

[Reviewer #1, Comment 7] Lines 697, 724, 802, 806: Please correct "CO2" to "CO_2".

[Author response] Corrected.

[Reviewer #1, Comment 8] Lines 776-777: Please add the DOI: https://doi.org/10.5751/ES-02412-130228.

[Author response] Added.

[Reviewer #1, Comment 9] Lines 839-840: Please add the DOI: https://doi.org/10.1111/nph.13949.

[Author response] Added.

Reviewer #2 (Remarks to the Author):
This manuscript examines how long-term climate change alters biological soil crust (biocrust) communities and how these shifts may feed back to local climate. Using a two-decade dryland climate manipulation experiment combined with multi-scale remote sensing, the authors assess the effects of experimental warming (+4 °C) and altered monsoonal rainfall on biocrust composition and associated ecosystem processes. They report a decline in late-successional moss-dominated crusts and an increase in early-successional lightly pigmented cyanobacteria, changes that are linked to reduced photosynthetic potential, lower surface moisture, and higher surface temperatures.

The study addresses an important and timely question because biocrusts cover a substantial fraction of global drylands and play key roles in regulating carbon uptake, soil moisture, and

surface energy balance. Understanding how climate-driven changes in biocrust communities influence land–atmosphere feedbacks is therefore critical, particularly as such processes are currently underrepresented in climate and ecosystem models.

[Author response] We sincerely thank the reviewer for their positive and constructive feedback, which has helped us to improve the manuscript. Please see below our point-by-point responses in blue text following each reviewer comment. Note, the line numbers in our responses are for the clean (non-tracked-change) version of the revised manuscript.

[Reviewer #2, Comment 1] The manuscript presents a robust experimental design, a valuable dataset, and statistically significant results, clearly documenting climate-driven shifts in biocrust community composition and associated changes in surface properties. To further strengthen the manuscript, it would be helpful to more explicitly highlight how these findings advance beyond previous studies, for example by clarifying the novel aspects of the long-term experiment, the integration of multi-scale remote sensing, or the quantification of feedbacks to surface temperature. Additionally, while the use of remote sensing to detect community shifts is compelling, the discussion could be expanded to illustrate the broader implications or potential applications of this approach, such as large-scale monitoring of dryland ecosystems or integration into predictive models. These clarifications would help readers better appreciate both the scientific contribution and practical relevance of the study.

[Author response] Excellent points. We now more clearly highlight the emerging potential for large-scale monitoring of dryland ecosystems in the main text (Lines 454 – 467) and we add a new final paragraph to the discussion that underscores the novelty of our findings and the uniqueness and potential adaptability of the approach (Lines 469 – 486). We include the revised text with changes italicized and underlined below for reviewer convenience.

Lines 454 – 467:
"Nevertheless, the consistent pattern of reduced photosynthetic potential and increased surface brightness from field to satellite measurements demonstrates the emerging potential to monitor biocrust community composition and function at high spatiotemporal resolution across global dryland region by leveraging next generation spaceborne platforms. *Fusion of high spatial resolution observations (e.g., WorldView-3, PlanetScope) that can capture the intricate spatial heterogeneity of biocrusts, with high spectral resolution observations (e.g., NASA EMIT, ESA EnMAP, Landsat Next) that can capture multiple functional traits and climate mitigation indices, including surface brightness and temperature, offers a promising way forward (Ji et al., 2026; Smith et al., 2019).* Large-scale biocrust monitoring efforts to quantify and build resilience in existing biocrust communities could provide innovative options for sustaining these organisms and reducing climate feedbacks, and remote sensing approaches offer powerful but currently under-utilized tools for vastly improving our capacity to assess, model, and make decisions for these foundational soil communities."

Lines 469 – 486:
"***Enhancement of global change experiments through the integration of remote sensing***
We demonstrate an approach in which we integrate multiscale remote sensing measurements (field, UAS, and satellite) within a long-term global change experiment to reveal a novel biocrust

degradation – climate warming amplification feedback that is likely already occurring across vast global dryland ecosystems. Further, our approach enabled a nuanced enhancement of previous efforts, which by focusing only on albedo, came to an opposite conclusion – expansion of early-successional light cyanobacteria and the loss of late-successional mosses could result in surface cooling due to albedo increases (Rutherford et al., 2017). Our more comprehensive view was achievable by the integration of multiscale remote sensing measurements over the optical and thermal regions of the electromagnetic spectrum, which revealed substantial increases in surface temperature in climate change treatment plots, despite major increases in light cyanobacteria fractional coverage and corresponding increases in surface brightness (Figure 3, 5). These findings underscore the need to monitor, restore, and protect late-successional biocrusts across global drylands for their significant role in ecosystem function and climate mitigation. The approach developed here could be easily adapted for application in a wide range of existing long-term global change experiments to complement traditional methods and extend the inferences gain from these critically important yet time-, labor-intensive, and expensive undertakings (Shiklomanov et al., 2019)."

[Reviewer #2, Comment 2] The connection between the observed compositional changes and the ecosystem's functional shifts is not fully articulated. Although the authors report changes in photosynthetic potential, surface moisture, and temperature, the ecological and biogeochemical consequences of these shifts are not discussed in detail. Expanding on how the documented community transitions translate into broader functional changes in dryland systems would clarify the study's significance and potential applications.

[Author response] We have now added more detail on the connection between the observed biocrust community compositional changes and ecosystem functional shifts (Lines 386 – 406). We feel this revised text has helped to underscore the study's significance. We include the revised text with changes italicized and underlined below for reviewer convenience.

Lines 386 – 406:
"Ultimately, the substantial reduction in biocrust *contributions to ecosystem function and* climate mediation with warming and warming + altered precipitation that we have identified here portends a novel and likely already occurring biocrust degradation – climate warming amplification feedback (Fig. 5). The warmer surface temperature of the lightly-pigmented compared to the darkly-pigmented biocrust communities, despite the higher albedo of the former, implies that increased sensible heat flux of the lightly-pigmented cyanobacteria more than compensate for their higher albedo. *We find an additional surface warming of ~0.3 °C for only a 10% shift in biocrust community composition – a shift that's likely already occurring across the 12% of the terrestrial land surface covered by biocrusts (Rodriguez-Caballero et al., 2018).* Additionally, reductions in photosynthetic capacity associated with the conversion from darkly-pigmented mosses to lightly-pigmented cyanobacteria *indicate significant ecosystem functional degradation and additional climate warming through reduced uptake of atmospheric CO_2 over space and time*. *Previous estimates suggest biocrusts account for roughly 0.58 Pg C per year of global gross primary productivity (Rodriguez-Caballero et al., 2018), which translates to a net global cooling potential of roughly ~0.08 °C by the end of the century (IPCC, 2021).* Our findings suggest that this indirect cooling pathway of biocrusts could be reduced by up to 66% with a large-scale shift towards lightly-pigmented cyanobacteria communities. Overall, given the clear dominance of warming

amplification feedbacks found here, it is likely that the net result of these direct (surface temperature) and indirect (carbon uptake) climate mitigation pathways will be *net warming, triggering continued ecosystem functional degradation and potential warming amplification*."

[Reviewer #2, Comment 3] The manuscript integrates measurements from several sensing platforms (handheld spectroradiometer, UAS multispectral/thermal imagery, and WorldView-3 satellite imagery), which is a strength of the study. However, it is not fully clear how measurements collected at these different spatial scales are made comparable. In particular, the plot-scale ground spectra (ASD FieldSpec measurements) represent very small sampling footprints and high spectral resolution (1 nm), whereas the satellite observations (WorldView-3) have a spatial resolution of ~1.24 m and only eight broad spectral bands. Similarly, the UAS imagery has centimeter-level spatial resolution and different spectral characteristics. While the manuscript states that pixels were aggregated to the plot scale for the satellite data, it remains unclear how the authors ensured comparability between these datasets in terms of spatial footprint, spectral band alignment, and radiometric consistency. The authors should clarify the scaling strategy used to link ground measurements, UAS observations, and satellite imagery. For example, it would be helpful to explain whether ground spectra were convolved to the spectral response functions of the airborne/satellite sensors, how spatial heterogeneity within plots was handled when aggregating pixels, and how uncertainties introduced by the scale mismatch were assessed. Without a clearer description of these steps, it is difficult to evaluate the robustness of the multi-scale remote sensing comparison.

[Author response] This is a very useful comment that led us to a deeper exploration of our scaling approach. First, we have now moved the WorldView-3 analysis figure to the main text (revised Fig. 4). We also added an additional methods section (Lines 671 - 681) and a new supplemental figure (revised Fig. S5) to more clearly explain our spectral aggregation and scaling approach. We finally include a new section in the results describing our findings (Lines 205 – 210) and new discussion of these findings (Lines 441 - 467). We include the revised figures and text with changes italicized and underlined below for reviewer convenience.

Lines 671 - 681:
*"**Plot-level comparison across spectral measurements***
We compared spectral surface reflectance estimates across products by aggregating the FieldSpec 3, UAS Micasense, and satellite WorldView-3 data to the plot-level and across shared spectral bands. FieldSpec plot-level median surface reflectance was aggregated using wavelength centroids and bandwidth to match the UAS MicaSense (10 bands) and the WorldView-3 (8 bands) spectral bands. UAS MicaSense and satellite WorlView-3 spectral data were compared across eight overlapping spectral bands that had similar wavelength centroids. We note that this relatively simple approach was used to evaluate whether consistent plot-level trends were detectable across commonly utilized platforms despite known uncertainties introduced due to differences in spectral response functions, bandwidth, radiometric properties, and spatial scale (Fig. S5)."

Lines 205 - 210:
"*We first evaluated our scaling framework by spectrally aggregating our field-scale hyperspectral data to match the UAS and WorldView-3 multispectral bands (see Methods). Comparison across*

field, UAS, and WorldView3 band estimates reveal positive relationships confirming the effectiveness of our scaling approach (Fig. S5). It is however worth noting that WorldView-3 showed a relatively dampened change in reflectance across spectral bands, with relatively high reflectance in the blue bands and relatively low reflectance in the red and near infrared bands."

Lines 441 - 467:
"As a proof-of-concept, we explored the potential to estimate plot-level chlorophyll and brightness index from very high spatial resolution satellite observations from WorldView-3 (1.24 m) (Fig. 4). *Importantly, as we scaled from field-, to UAS-, to satellite-based estimates, the magnitude of the change in each climate regulation trait was notably reduced. For instance, the change in surface brightness was estimated to be +14.1%, +7.5%, and +2.3% for field-, UAS-, and satellite-based estimates, respectively (Fig. 3, 4). This is likely due to increasing edge effects from untreated pixels as the spectral and spatial resolution of the measurements became coarser from the field to UAS to satellite scale (Fig. S5). Additionally, uncorrected atmospheric issues could be increasingly contributing to uncertainty or introducing bias from the field to UAS to satellite scale. Chlorophyll indices experienced even larger signal reduction: -6.3%, -3.3%, -1.5% at the field-, UAS-, and satellite-scale, respectively. Chlorophyll indices are likely further biased by Raleigh scattering, which has a larger impact on visible compared to near-infrared wavelengths and increases in importance with atmospheric path length from UAS to satellite measurements (Fig. S5).* Nevertheless, the consistent pattern of reduced photosynthetic potential and increased surface brightness from field to satellite measurements demonstrates the emerging potential to monitor biocrust community composition and function at high spatiotemporal resolution across global dryland regions by leveraging next generation spaceborne platforms. Fusion of high spatial resolution observations (e.g., WorldView-3, PlanetScope) that can capture the intricate spatial heterogeneity of biocrusts, with high spectral resolution observations (e.g., NASA EMIT, ESA EnMAP, Landsat Next) that can capture multiple functional traits and climate mitigation indices, including surface brightness and temperature, offers a promising way forward (Ji et al., 2026; Smith et al., 2019). Large-scale biocrust monitoring efforts to quantify and build resilience in existing biocrust communities could provide innovative options for sustaining these organisms and reducing climate feedbacks, and remote sensing approaches offer powerful but currently under-utilized tools for vastly improving our capacity to assess, model, and make decisions for these foundational soil communities."

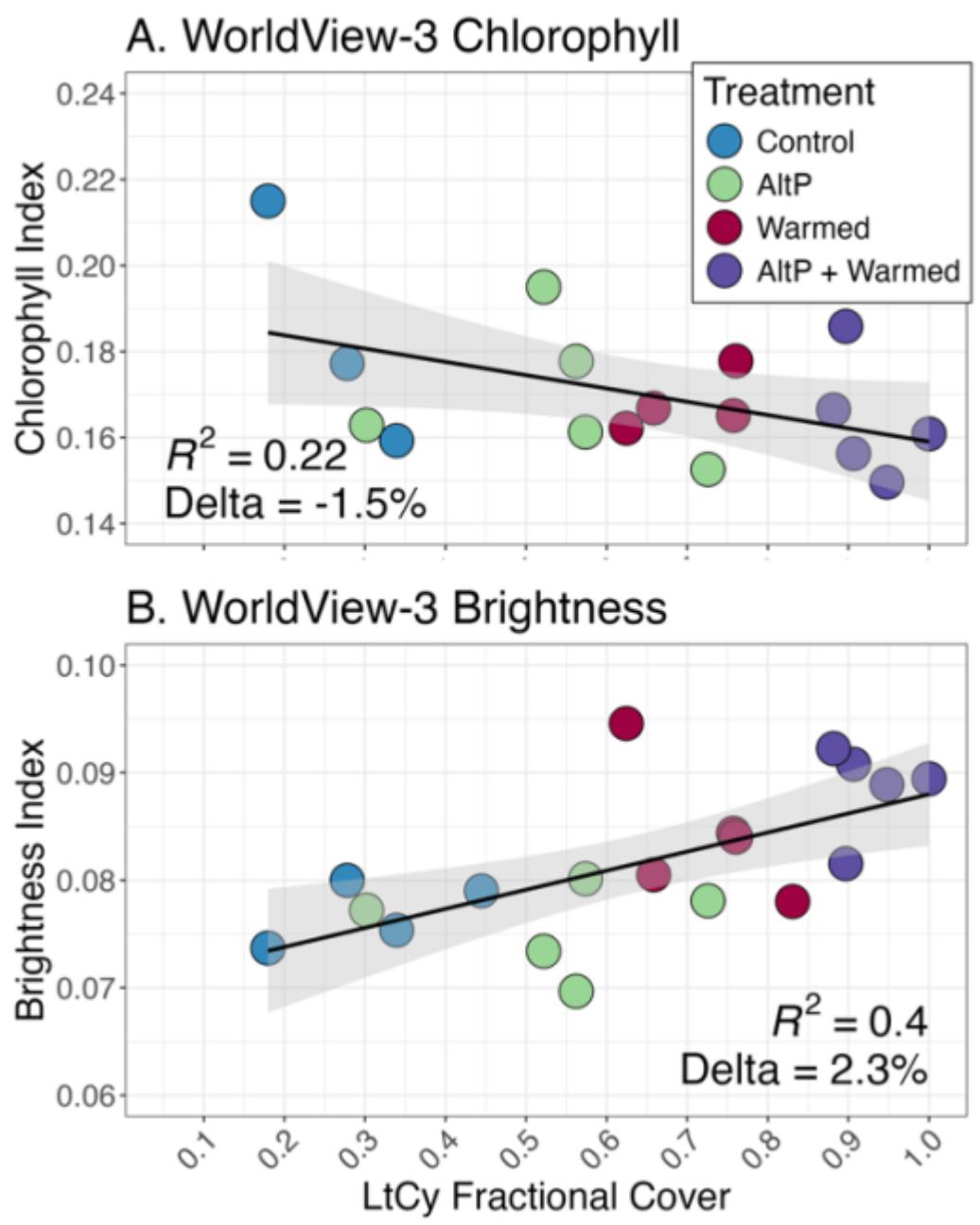

Figure 4. The relationship between WorldView-3 climate regulation indices and light cyanobacteria (LtCy) fractional cover across all experimental plots. *Satellite observations used to derive the satellite-based chlorophyll (A) and brightness index (B) were from a WorldView-3 scene that was collected during the field campaign. Light cyanobacteria fractional cover was estimated as the ratio of light cyanobacteria cover to the sum of total BFT cover (e.g., the sum of light cyanobacteria, dark cyanobacteria, lichen, and moss cover). Each circle in the plot represents a single plot and is colored by treatment: control (Control) altered precipitation (AltP), warming (Warmed), and altered precipitation and warming combined (AltP + Warmed). R2 represents the proportion of the variance for a given climate regulation index that's explained by light cyanobacteria fractional cover. Delta represents the percentage change for a given climate regulation index per 0.1 change in light cyanobacteria fractional cover.*

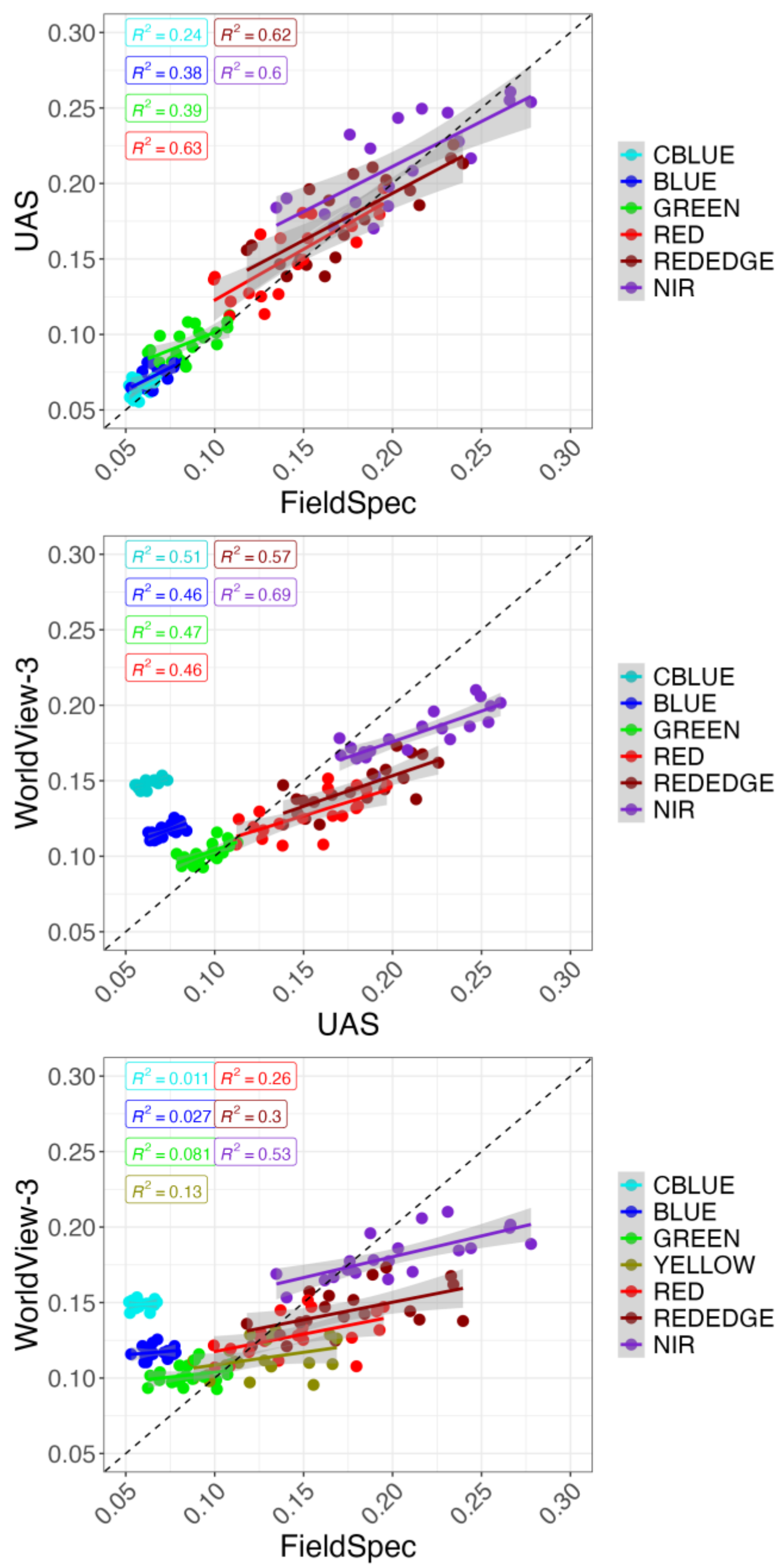

Figure S5. The relationship between multi-scale remotely sensed surface reflectance estimates across all experimental plots. ASD FieldSpec versus uncrewed aerial system (UAS) estimates of surface reflectance across all plots and shared spectral bands (A). UAS versus satellite WorldView-3 estimates of surface reflectance across all plots and shared spectral bands (B). ASD FieldSpec versus satellite WorldView-3 estimates of surface reflectance across all plots and shared spectral bands (C). R^2 *represents the proportion of the variance for a given spectral band that's explained by the independent variable.*

[Reviewer #2, Comment 4] As satellite observations can reproduce or approximate plot-scale patterns observed in the field (which still needs to be validated for comparison, see my previous comment), the manuscript should further explore the temporal potential of satellite data.

Specifically, once the relationship between plot measurements and satellite reflectance is established, historical satellite archives could be used to examine how these plots may have changed over time. Such an analysis could offer valuable context for the experimental results by placing the observed community shifts within a longer-term perspective and evaluating whether the detected biocrust changes align with broader trends observable at the site level. Even a preliminary analysis of such time series could significantly strengthen the manuscript by demonstrating the potential for long-term monitoring of biocrust dynamics from space and by emphasising the practical relevance of satellite remote sensing beyond the timeframe of field experiments.

[Author response] Unfortunately, due to the experimental infrastructure that would influence the spectral signal recorded by satellite (i.e., lamps positioned over each plot), we are unable to go back in time and utilize high quality satellite observations of the experiment. However, to better demonstrate the potential for scaling with satellite observations, we have moved the WorldView-3 results to the main paper (revised Fig. 4). We also now directly point out that plot infrastructure was removed during the WorldView-3 overpass during our study period, which enabled high quality plot-level observations (Lines 567 – 575). We include the revised figures and text with changes italicized and underlined below for reviewer convenience.

Lines 567 – 575:
"We additionally incorporated an 8-band multispectral WorldView-3 image from February 12, 2022 to explore the potential of monitoring treatment effects at the plot scale using very high resolution (1.24 m) satellite data. *Importantly, the warming treatment infrastructure was removed during the WorldView-3 overpass so that the satellite had a clear view of all study plots and was unimpacted by plot infrastructure.* The experimental site was ideally suited for the incorporation of multi-scale remote sensing techniques given the uniformity of soil type and texture across plots and the overall size and location of the plots within the site. We describe these collected datasets in more detail below."

[Reviewer #2, Comment 5] Figure 1a: The location of the study site is not immediately clear. Consider providing an improved map with clearer boundaries, labels, and scale to help the reader orient themselves.

[Author response] We have now improved Fig. 1 by enlarging the site marker and labeling the states that make up the Colorado Plateau. This revised figure is included below for reviewer convenience.

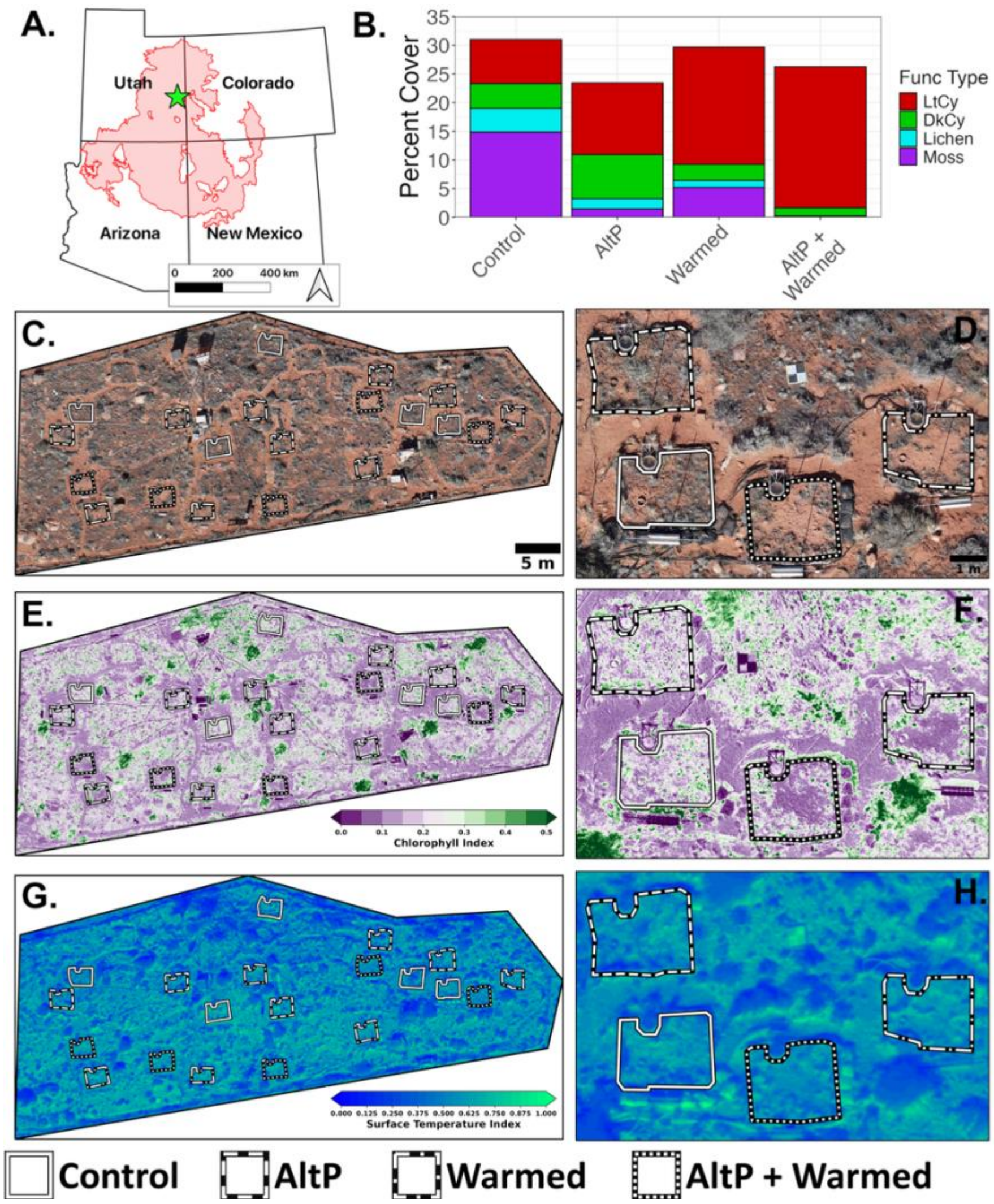

Figure 1. The long-term biocrust climate manipulation experiment located near Castle Valley, Utah (38.67° N, 109.42° W) on the Colorado Plateau. Map showing location of the study site (green star) and Colorado Plateau Ecoregion (red polygon) spanning U.S. states Colorado, Utah, Arizona, and New Mexico (A). Bar plot showing the percent cover of the major biocrust functional types (BFTs; lightly-pigmented cyanobacteria (LtCy), darkly-pigmented cyanobacteria (DkCy), lichen, and moss) and the dominant shift from mosses in the control plots to lightly-pigmented cyanobacteria in the warmed plots (B). Uncrewed aerial system (UAS)-based red, green, blue (RGB) image of the study site (C) and a zoomed in aerial view of the eastern most plots representing the four experimental treatments (treatments and associated line types are listed below the figure: Control, Altered Precipitation (AltP), Warmed, and AltP and Warmed (AltP + Warmed treatments) (D). UAS-based Chlorophyll Index image of the study site (E) and a zoomed in aerial view of the eastern-most plots representing the four experimental treatments (F). UAS-based Surface Temperature Index image of the study site (G) and a zoomed in aerial view of the eastern most plots representing the four experimental treatments (H). The figure shows how RGB, Chlorophyll, and Surface Temperature indices were all able to capture aspects of the biocrust community composition changes that were caused by the warming and altered precipitation treatments.

[Reviewer #2, Comment 6] Lines 166–167: The text states that only one cyanobacterial BFTs showed no differences between treatments for all indices, but Fig. S3 is not fully reflected in this description (both cyanobacterial BFTs show no differences). Consider revising the text to more accurately summarize what the figure shows, so that readers can clearly see the correspondence between the results and the figure.

[Author response] We thank the reviewer for catching this oversight. We have now corrected this issue and Fig. S3 is now consistent with the statistics reported in the revised text and supplemental tables (Table S3-S6).

Experimental evidence of a biological soil crust degradation climate warming amplification feedback

Response to Reviewers

Editor (Remarks to the Author):

Your manuscript titled "Experimental evidence of a biological soil crust degradation climate warming amplification feedback" has now been seen by 2 reviewers, and we include their comments at the end of this message. They find your work of interest, but some important points are raised. We are interested in the possibility of publishing your study in Communications Earth & Environment, but would like to consider your responses to these concerns and assess a revised manuscript before we make a final decision on publication.

[Author response] We greatly appreciate the constructive comments from the editor and reviewers, which we closely followed to thoroughly revise our manuscript. Please see below our point-by-point responses in blue text following each comment. Note, the line numbers in our responses are for the clean (non-tracked-changes) version of the revised manuscript.

Reviewer #1 (Remarks to the Author):
This research provides crucial theoretical foundations and methodological support for refining biocrust feedback mechanisms in Earth system models, formulating dryland ecological conservation strategies, and establishing a global biocrust dynamic monitoring system. I have checked all the comments from the two reviews to the manuscript and found that the authors have carefully considered all the comments. The manuscript is very strong now. However, there still have a question that the biological soil crust communities at different succession stages respond differently to climate change. Therefore, what are the response mechanisms of the biological soil crusts at different succession stages to climate change? What are the similarities and differences? Especially in Figure 5, I think this concept diagram is a bit simplistic and lacks the description of the process.

[Author response] First, we sincerely thank the reviewer for their positive and constructive feedback. We have now added a paragraph to the main text that explains the different response mechanisms of the biocrust at different succession stages to climate change (Lines 324-340). Additionally, we have revised Figure 5 to better describe these different response mechanisms. We have included the revised text and figures with changes italicized and underlined below for reviewer convenience.

Lines 324 – 340:
"*Major shifts from a late-successional, darkly-pigmented to an early-successional, lightly-pigmented biocrust community were observed across warmed and warmed + altered precipitation plots. For instance, in the warmed + altered precipitation treatment, fractional coverage of late-successional mosses decreased by ~27% and the fractional cover of early-successional, lightly-pigmented cyanobacteria increased by ~36%. Moss and to a lesser extend*

lichen mortality due to warming and/or altered precipitation has been previously explained by a combination of interacting factors including: i) more rapid desiccation with higher temperatures resulting in reduced carbon uptake per rainfall event; ii) increased respiration rates with high temperatures resulting in an increase in baseline carbon losses; iii) stalled photosynthesis with altered precipitation whereby the cost incurred with the initiation of metabolic activity at the onset of hydration can become greater relative to the caron gains (Ferrenberg et al., 2015; Phillips et al., 2022; Reed et al., 2012). Together these factors can drive an increasing carbon deficit that can ultimately lead to carbon starvation and mortality of mosses and lichens. Conversely, early-successional, lightly-pigmented cyanobacteria are relatively less sensitive to warming and/or altered precipitation, which explains their expanded coverage in these plots (Ferrenberg et al., 2015; Phillips et al., 2022; Reed et al., 2012)."

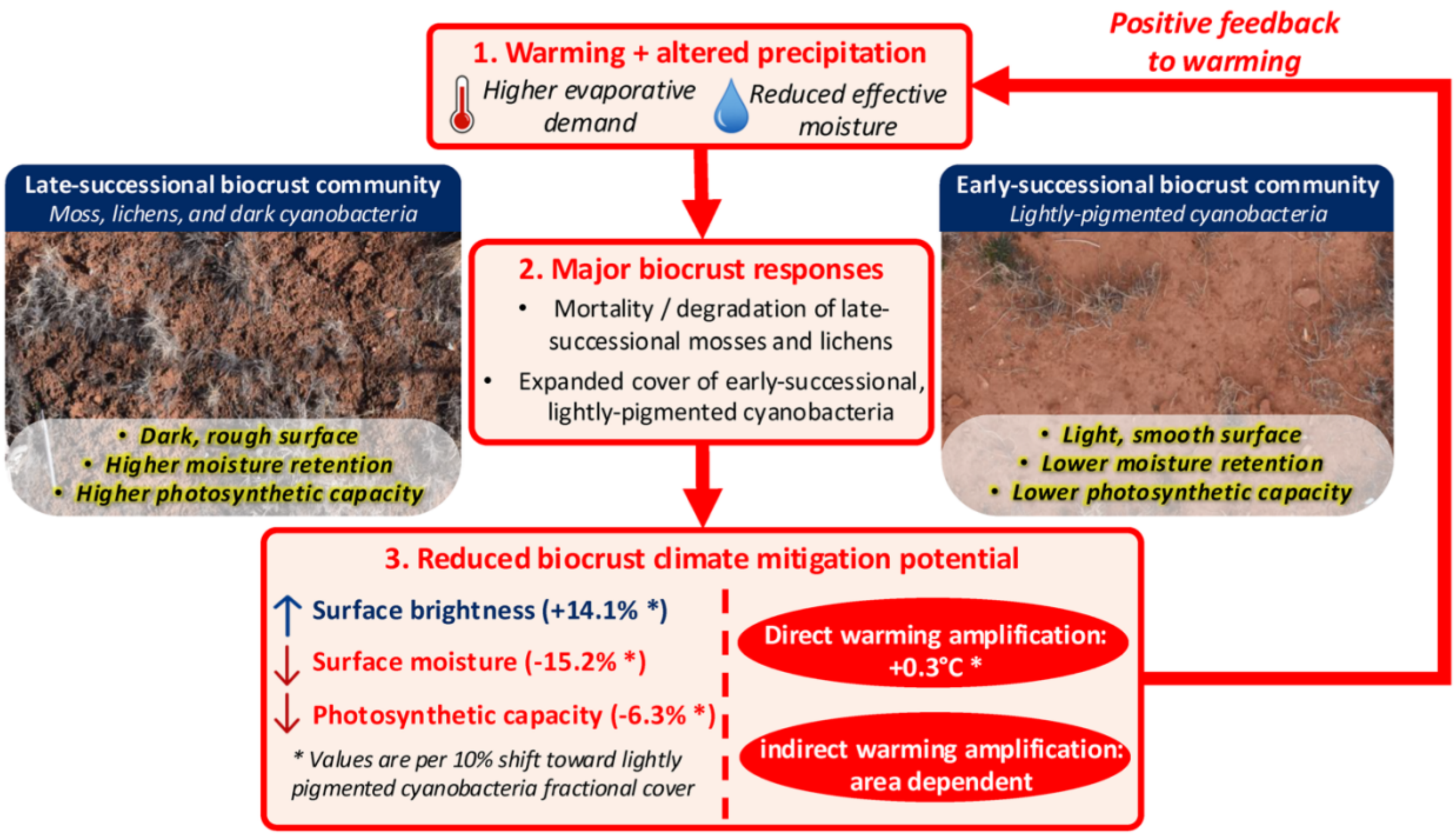

Figure 5. Conceptual figure illustrating a potential biocrust degradation climate warming amplification feedback. Warming + altered precipitation leads to moss and lichen degradation / mortality, most likely due to an increasing carbon deficit and ultimately carbon starvation; whereas drought and heat tolerant early-successional, lightly-pigmented cyanobacteria coverage expands. This shift in biocrust function and community composition can lead to a substantial reduction in climate mitigation potential and both a direct and indirect warming amplification feedback.

Reviewer #2 (Remarks to the Author):
I would like to thank the authors for their thorough revision. The MS has greatly improved. I don't have further specific comments.

[Author response] We sincerely thank the reviewer for their constructive feedback and positive assessment of the revised manuscript.

Minor comment:
Fig. 5: The text in red in the picture (left picture) is hard to read. Please consider using another colour.

We have significantly revised Figure 5 and improved the readability of all text. We have included the figure revisions below for reviewer convenience.

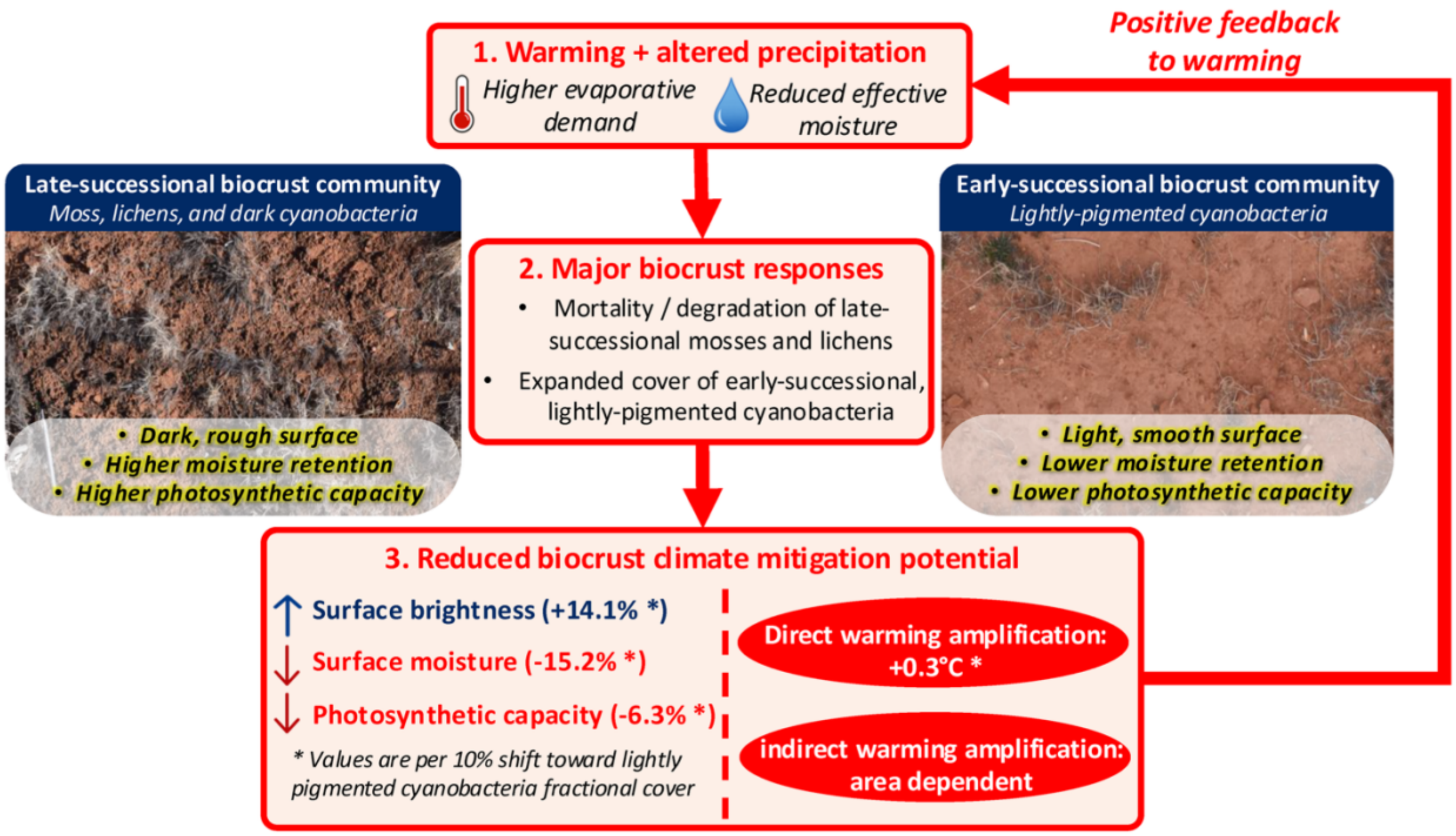

Figure 5. Conceptual figure illustrating a potential biocrust degradation climate warming amplification feedback. Warming + altered precipitation leads to moss and lichen degradation / mortality, most likely due to an increasing carbon deficit and ultimately carbon starvation; whereas drought and heat tolerant early-successional, lightly-pigmented cyanobacteria coverage expands. This shift in biocrust function and community composition can lead to a substantial reduction in climate mitigation potential and both a direct and indirect warming amplification feedback.

Reviewer Report

Journal: Communications Earth & EnvironmentManuscript

Title: Experimental evidence of a biological soil crust degradation climate warming amplification feedback

Summary of the Manuscript

This study investigates the impact of biological soil crust (biocrust) degradation—driven by experimental warming and physical disturbance (trampling)—on land surface albedo and land surface temperature (LST) on the Colorado Plateau. By utilizing high-resolution hyperspectral drone imagery , the authors demonstrate that the loss of late-successional biocrusts (lichen and moss) exposes darker bare soil, leading to a reduction in albedo (0.05–0.14) and a subsequent increase in modeled LST (0.61–1.63 °C). The authors calculate the localized radiative forcing of this degradation (3.6 ± 2.2 Wm^2) and propose a positive feedback loop wherein warming-induced biocrust loss amplifies surface heating, potentially exceeding the local forcing effects of elevated atmospheric CO^2.

General Assessment

The manuscript addresses a critical and underappreciated mechanism in dryland eco-climatology: the biophysical feedback of biocrust degradation. The experimental design, combining long-term manipulation (warming/trampling) with cutting-edge remote sensing (hyperspectral UAV), is innovative and robust. The topic is timely and fits well within the scope of Communications Earth & Environment, as it bridges ecology, remote sensing, and climate science.

However, while the central premise is compelling, there are several key areas regarding the energy balance modeling, spatial scaling assumptions, and the framing of the "feedback" mechanism that require rigorous clarification and revision before publication. The comparison between local surface radiative forcing and global greenhouse gas forcing needs to be handled with greater nuance to avoid misleading comparisons.

Major Comments

1. **Validation of Modeled Land Surface Temperature (LST).** The authors derive LST changes primarily based on albedo changes using the Stefan-Boltzmann law, assuming that albedo is the dominant driver. While albedo is undoubtedly critical, LST is also strongly influenced by emissivity and sensible/latent heat fluxes.

Emissivity Differences: Biocrusts (rough surface, complex microstructure) and bare soil (smoother, mineral composition) likely have different thermal emissivities. The manuscript does not clarify if a constant emissivity was used or if spectral emissivity was measured. A change in emissivity could dampen or amplify the LST signal.

Evaporative Cooling: While drylands are water-limited, biocrusts affect soil moisture retention. Does the degradation to bare soil alter evaporation rates (and thus latent heat flux) enough to impact LST?

Validation: Did the authors collect any in situ thermal measurements (e.g., IR thermometers or thermal camera on the drone) to validate the *modeled LST*? If physical temperature data exists, it must be shown to corroborate the modeled *ΔLST*. If not, a sensitivity analysis regarding emissivity and non-radiative fluxes is necessary.

2. **Spatial Scaling and Soil Background Heterogeneity.** The study extrapolates the findings from the experimental plots to the entire Colorado Plateau to estimate regional radiative forcing. This extrapolation assumes that the optical contrast between "biocrust" and "bare soil" observed at the Canyonlands site is representative of the entire region.

Soil Heterogeneity: The Colorado Plateau contains a diverse array of soil parent materials, ranging from bright gypsiferous soils to dark basaltic soils. In areas with naturally dark soils, biocrust loss might not lead to a significant albedo decrease (or could even increase albedo if the lichen is dark). The authors should discuss how soil background color variability might affect their regional forcing estimates.

Biocrust Types: The study focuses on high-biomass lichen/moss crusts. However, vast areas are covered by light cyanobacterial crusts. The albedo difference between light cyanobacterial crusts and bare soil is much smaller than that of lichen crusts. The regional extrapolation should explicitly account for the proportion of late-successional vs. early-successional crusts to avoid overestimating the feedback.

3. **Contextualizing Radiative Forcing: Local vs. Global.** The abstract and conclusion highlight that the biocrust forcing ($3.6 Wm^{-2}$) exceeds the forcing of doubling CO_2. While mathematically correct at the surface level locally, this comparison can be misinterpreted by a broad audience.

Mechanism Distinction: GHG forcing works by trapping outgoing longwave radiation (affecting the whole atmospheric column globally), whereas albedo change is a surface forcing

(affecting local surface energy budget).

Recommendation: The authors should explicitly distinguish between "local surface heating" and "global climate forcing." While the local impact is indeed massive and critical for local microclimates and organism survival, framing it as "exceeding CO_2" without qualification might imply it has a stronger driver on global circulation, which is not the claim. Please refine the language to emphasize "localized heating amplification" rather than a direct equivalent to GHG forcing.

4. **The "Feedback" Loop Dynamics.** The conceptual model in Figure 5 describes a "warming amplification feedback.

"Step-Change vs. Continuous Feedback: Is this a continuous feedback loop (runaway effect) or a state-change feedback? Once the crust is gone (degraded to bare soil), the albedo hits a "floor," and the feedback stops. The manuscript should clarify the limits of this feedback. It amplifies warming during the transition from crust to soil, but does it continue once the transition is complete?

Recovery Hysteresis: The paper mentions "recovery". Given the slow growth of lichens, is this feedback effectively irreversible on human timescales? Discussing the hysteresis (tipping point) would strengthen the ecological implications.

Minor Comments

1. Figure 1 (Conceptual Model): The diagram is helpful, but the distinction between "Actual Future" and "Projected Future" could be clearer. Perhaps use "Without Feedback" vs. "With Feedback" for clarity.

2. Hyperspectral Methodology: The text mentions using a "Nano-Hyperspec" sensor. Please specify the spectral resolution and range used for the albedo integration. Did you integrate over the full solar spectrum (including SWIR) or just the VNIR range of the sensor? If only VNIR (400-1000nm), this might underestimate albedo if soil/crust differences are prominent in the SWIR. Please address this limitation.

3. Treatment Effectiveness: In Figure 3, the "Warming" treatment shows a significant reduction in lichen cover, but "Warming + Trampling" is even more severe. It would be interesting to see if there is an interaction effect. Is the crust more susceptible to physical damage when heat-stressed?

4. Reference Format: Ensure all citations in the text match the bibliography.

5. Terminology: The term "amplification feedback" is slightly redundant; "positive feedback" is the standard term. Consider simplifying the title to "...degradation creates a positive climate feedback."

6. Methodology Clarification: It is excellent that the warming lamps were turned off/removed before measurement. Please emphasize this earlier in the abstract or results to prevent readers from assuming the temperature difference is a direct artifact of the lamps.

7. Figure 4: The conceptual diagram implies a "runaway" feedback loop. Does this feedback stop once the moss is completely gone? Clarify the limits of this feedback loop.

8. Typos: Line 2370: "median = 216.2°C" is clearly a typo for 16.2°C. Please check all values.

Recommendation

Publish with Major Revisions.The data is high quality and the finding is significant. Addressing the energy balance validation and adding nuance to the regional scaling/forcing comparison will make this a landmark paper for dryland ecology and climate change research.
